# Microanatomy of Adult Zebrafish Extraocular Muscles

**DOI:** 10.1371/journal.pone.0027095

**Published:** 2011-11-23

**Authors:** Daniel S. Kasprick, Phillip E. Kish, Tyler L. Junttila, Lindsay A. Ward, Brenda L. Bohnsack, Alon Kahana

**Affiliations:** Department of Ophthalmology and Visual Sciences, Kellogg Eye Center, University of Michigan, Ann Arbor, Michigan, United States of America; National Institutes of Health/NICHD, United States of America

## Abstract

Binocular vision requires intricate control of eye movement to align overlapping visual fields for fusion in the visual cortex, and each eye is controlled by 6 extraocular muscles (EOMs). Disorders of EOMs are an important cause of symptomatic vision loss. Importantly, EOMs represent specialized skeletal muscles with distinct gene expression profile and susceptibility to neuromuscular disorders. We aim to investigate and describe the anatomy of adult zebrafish extraocular muscles (EOMs) to enable comparison with human EOM anatomy and facilitate the use of zebrafish as a model for EOM research. Using differential interference contrast (DIC), epifluorescence microscopy, and precise sectioning techniques, we evaluate the anatomy of zebrafish EOM origin, muscle course, and insertion on the eye. Immunofluorescence is used to identify components of tendons, basement membrane and neuromuscular junctions (NMJs), and to analyze myofiber characteristics. We find that adult zebrafish EOM insertions on the globe parallel the organization of human EOMs, including the close proximity of specific EOM insertions to one another. However, analysis of EOM origins reveals important differences between human and zebrafish, such as the common rostral origin of both oblique muscles and the caudal origin of the lateral rectus muscles. Thrombospondin 4 marks the EOM tendons in regions that are highly innervated, and laminin marks the basement membrane, enabling evaluation of myofiber size and distribution. The NMJs appear to include both *en plaque* and *en grappe* synapses, while NMJ density is much higher in EOMs than in somatic muscles. In conclusion, zebrafish and human EOM anatomy are generally homologous, supporting the use of zebrafish for studying EOM biology. However, anatomic differences exist, revealing divergent evolutionary pressures.

## Introduction

**Figure 1:**
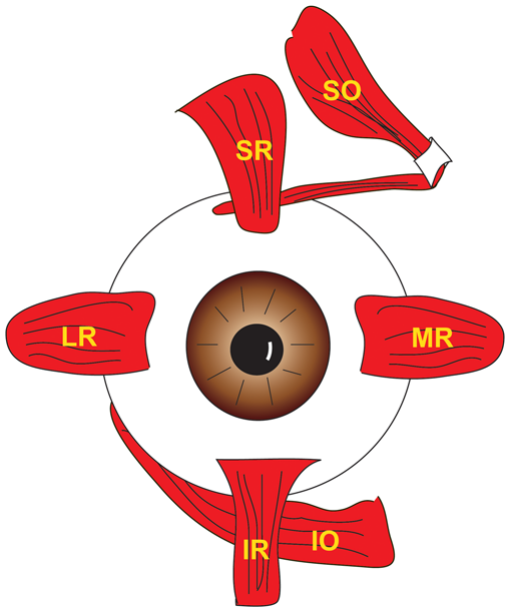
Illustration of human eye showing 6 EOMs inserting on the globe in what is referred to as the Spiral of Tillaux.

Zebrafish and humans both utilize six highly specialized extraocular muscles (EOMs) per eye to control the precise pursuit and saccade movements required to track moving items and maintain stable images on the retina for high acuity vision. In humans, five of the six muscles – inferior rectus (IR), superior rectus (SR), lateral rectus (LR), medial rectus (MR), and superior oblique (SO) - originate at the Annulus of Zinn, a common tendinous ring of fibrous tissue that surrounds the optic nerve, ophthalmic artery, and ophthalmic vein at their entrance through the apex of the orbit. The sixth muscle, inferior oblique (IO), has a separate origin point on the orbital side of the bony maxilla at the anterior inferomedial strut. Each of these muscles has a distinct insertion site on the globe (Figure 1) and generates a unique primary rotation of the eye when acting alone. Additionally, each muscle has secondary and tertiary influences over eye movement when combined with action from one or more of the other six EOMs. The specific eye movements elicited by each muscle or group of muscles is dictated by the anatomical position of the EOM origin sites within the bony orbit, the functionality of connective tissue pulleys, the insertion site positions of the EOMs on the eye, and the rotational position of the eye which modifies the primary tension vector generated by any given muscle. Highly coordinated contraction of the proper EOMs at the proper time allows humans to achieve binocular vision. This mode of vision provides stereoptic cues for depth perception and object size determination, but limits the range of the cumulative visual field.

Human EOM is divided into two layers with characteristic innervations, fiber types [1], [2], metabolism [3], and gene expression profiles [4], [5], [6]. The inner global layer (GL) inserts on the eye and the similarly sized outer orbital layer (OL) inserts on a connective tissue ring forming the EOM pulley system. The OL positions the EOM pulley along individual rectus muscles to change the position of the functional origin. The OL and GL are also distinguished from each other by a 2-fold greater density of multiply innervated fibers (MIFs) observed in OL muscle [1]. Both the GL and OL are dominated by singly innervated fibers (SIF), similar to skeletal muscle, but differences in neuromuscular junction (NMJ) distribution patterns have been observed between EOM and limb muscle in several animal models [7]. Changes in NMJ frequency or distribution can serve as important markers for neuromuscular disease [8], [9] and are an important component of EOM anatomy. The unique functional and morphological characteristics of EOM can be attributed at least partially to its unique embryonic origin involving interaction between cranial mesoderm and migrating neural crest cell populations [10], [11], [12], [13], [14].

Zebrafish eyes are positioned laterally on the head providing a field of view that surpasses that of humans but leaves fish with a limited area of overlapping visual fields. The nomenclature of the six EOMs in zebrafish remains the same as in humans and the overall anatomic organization of the muscles within the orbit shows distinct similarities as well. In 1996, Stephen Easter reported on the organization of the EOMs within the orbit of a 96-hour post fertilization (hpf) embryo [15], but there have been no systematic studies published on the comparative anatomy of adult zebrafish EOMs. The present study highlights the remarkable similarity between zebrafish and human EOM gross and microscopic anatomy through a detailed exploration of the adult zebrafish orbit both in vivo and using two-dimensional cross section analysis. We propose that adult zebrafish can serve as a useful model for studying EOM structure and function.

## Materials and Methods

### Zebrafish (*Danio rerio)* Rearing

All animal work was performed ethically and in compliance with the ARVO Statement for the Use of Animals in Ophthalmic and Vision Research, and approved by the University of Michigan Committee on the Use and Care of Animals, protocol 10205. The *Tg(*α *-actin::EGFP)* fish line was a generous gift from Dr. Simon Hughes, Kings College London, United Kingdom. Sexually mature adult (4-18 month old) wild type (WT) and transgenic *Tg(*α*-actin::EGFP)* zebrafish were raised per standard protocol at 28°C with a 14-h light/10-h dark alternating cycle. *Tg(*α*-actin::EGFP)* lines express EGFP under the control of the muscle-specific α-actin promoter, enabling visualization of EOMs *in vivo* [16], [17].

### Specimen Processing and Microscopy

Adult *Tg(*α*-actin::EGFP)* fish were anesthetized in 0.05% tricaine and placed on a moist viewing platform created by placing roughly 10 lab tissues into a 0.5 cm deep, 9.5 cm diameter petri dish lid and saturating with 0.05% tricaine diluted in fish system water. Fish were laid flat on one side for visualization of the MR and LR and were propped up against moistened folded paper towel with the dorsal side up to image the SR and SO and with the ventral side up to image the IR and IO. A micromanipulator equipped with a fine point blunt probe was used to gently rotate and hold the eye in the proper position for muscle exposure and image capture. A Leica MZ16FA stereomicroscope with a Leica DFC 295 camera (Leica Microsystems CMS GmbH, Wetzler Germany) was used to capture fluorescent images highlighting EGFP-expressing EOM. Images were processed using Photoshop CS5 (Adobe Systems, San Jose, California) and Leica Application Suite Advanced Fluorescence (LAS AF) software.

Fish to be used for DIC gross anatomy mosaics were euthanized, decapitated several millimeters behind the gills using a clean razor blade, and fixed in 4% paraformaldehyde (PFA) for 2 hours at room temperature. Decalcification was achieved by gently shaking in 5% nitric acid for 10 minutes, washing in distilled deionized water twice for 5 minutes, quenching in dilute ammonium hydroxide (10 drops of pure ammonium hydroxide added to 200 ml distilled deionized water), and then additional washing in water for 10–20 minutes. These samples were gently shaken overnight at 4°C in 5% sucrose in 0.1 M phosphate buffer and then changed over to 20% sucrose in 0.1 M phosphate buffer and gently shaken at 4°C overnight once again. Specimens were placed into an OCT filled mold and frozen in a dry-ice cooled ethanol bath. Specimens were sectioned at 12 microns, mounted on slides, dried at RT for 30 min, and rinsed twice in PBS for 5 minutes to remove excess OCT. Permanent coverslips were placed using ProLong Gold Antifade Reagent (Invitrogen).

Fish to be used for immunohistochemistry or α-bungarotoxin (BTX) staining were euthanized, briefly dipped in Shandon M-1 embedding matrix (Thermo Shandon, Pittsburgh, PA), and snap frozen by dropping the head into a liquid nitrogen bath and allowing 5–10 seconds for freezing to occur. Specimens were transferred to dry-ice chilled conical tubes and stored at −80°C until sectioning. Twelve micron-thick sections were cut and slides were kept on dry ice or in a −80°C freezer until ready for staining and imaging.

Images were obtained using a Leica DM6000 B microscope with Leica DFC295 (DIC) and Hamamatsu ORCA (epi-fluorescence) cameras (Hamamatsu Photonics, Hamamatsu City, Japan), using Leica LAS and LAS AF software. Mosaics were created by capturing images of the entire desired field of view by moving the microscope stage by hand and ensuring roughly 25% overlap between consecutive images. Images were merged using the automated “photomerge” function in Adobe Photoshop CS5. Individual muscles were shaded on coronal and transverse DIC mosaics using the Photoshop paintbrush tool. High magnification fluorescent data obtained with the 40x and 63x objectives were gathered as z-stack images, then deconvolved and 3D-projected using LAS AF software.

Whole muscle mosaic images of α-BTX labeled NMJ's were obtained using a Leica SP5 confocal imaging microscope with an automated stage for coordinated image tile capture. Leica LAS AF software was used to assemble individual tiles into complete mosaics and z-stacks were converted into single layer images using the LAS AF 3D projection tool.

### Immunohistochemistry

Slides were warmed for 2–3 minutes at room temperature then placed into 50 ml of ice-cold methanol for 10 minutes to fix the tissue. Slides were washed once for 5 minutes in PBS, placed in blocking solution (5% goat serum in PBS +0.2% Tween) for 30 minutes, and then washed in PBS + 0.2% Tween (PBST) 4 times for 5 minutes. Slides were incubated in a humid chamber for 2 hours at room temperature in primary antibody (rabbit polyclonal anti-laminin, Sigma Aldrich, St. Louis, MO; rabbit polyclonal anti-thrombospondin-4 (Thbs-4), Santa Cruz Biotechnology Inc., CA) diluted to 1∶200 in PBST + 2% goat serum. Slides were again washed 4 times for 5 minutes in PBST and then incubated in the dark with Alexafluor 647-conjugated goat anti-rabbit secondary antibody (Invitrogen) diluted 1∶1000 in PBS + 0.2% Tween. Slides were washed again and nuclei were stained with Hoechst (Anaspec Inc., San Jose, CA) for 2 minutes at a concentration of 2 µM in PBS. A final wash was done with PBS followed by permanent coverslipping with ProLong Gold Antifade Reagent. Slides were stored long term at 4°C in the dark.

### Neuromuscular Junction Labeling and Density Quantification

Slides were washed for 5 minutes in PBST to remove excess embedding medium then incubated for 2 hours in 50 ml of cold 1 µg/ml Alexafluor 488-conjugated α–BTX; (Invitrogen) diluted in PBS + 2 µm sodium azide. Slides were washed for 5 minutes in PBS and nuclei were stained with Hoechst nuclear stain for 2 minutes at a concentration of 2 µm in PBS. A final 5-minute wash in PBS was followed by application of ProLong Gold Anti-Fade Reagent and coverslipping. Slides were stored long term at 4°C in the dark. For double-staining, the α-BTX binding was performed immediately following the washing of the secondary antibody. After image capture, muscle surface area was calculated using ImageJ software version 1.44 (National Institute of Health, Bethesda, Maryland). Total surface area of α–BTX – labeled NMJs was calculated using the ImageJ “particle analysis” tool to automatically trace and calculate the total combined surface area of NMJs. Mouse anti-synaptotagmin antibodies (znp1; Zebrafish International Resource Center, Eugene, OR) were used at 1∶100 dilution to identify presynapses, and double-staining experiments with znp1 and α–BTX enabled evaluation of the morphology and distribution of the NMJs.

## Results

### EOM Anatomy - *In vivo* Microscopy

**Figure 2:**
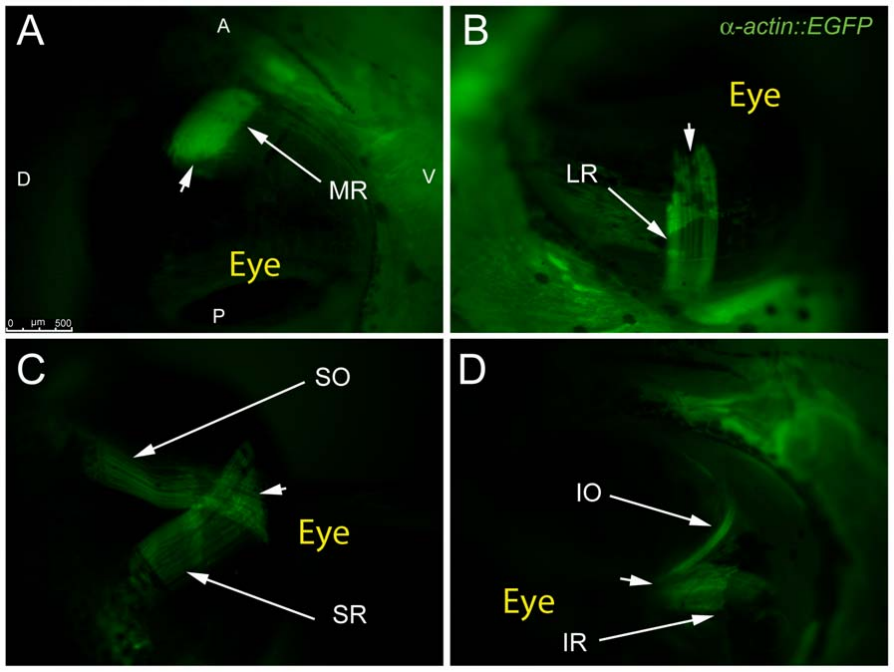
Adult transgenic zebrafish expressing GFP under the control of the *α-actin* muscle protein promoter allow for clear visualization of all 6 EOMs using epifluorescent stereomicroscopy. The MR and LR (A,B) are shown running parallel to the long axis of the fish and inserting on the anterior and posterior sides of the eye respectively. The SO and SR course from their respective rostral and caudal origin points to insert on the dorsal side of the eye with significant overlap of fibers (C). The IO and IR mirror the SO and SR as they insert onto the ventral side of the eye. Arrowheads mark scleromuscular insertion sites located at the scleral-corneal (SC) junction. Muscle origins are deep within the orbit and are not visible. The anterior (A), posterior (P), dorsal (D), and ventral (V) directions are noted on frame A and apply to frames B–D.

**Figure 3:**
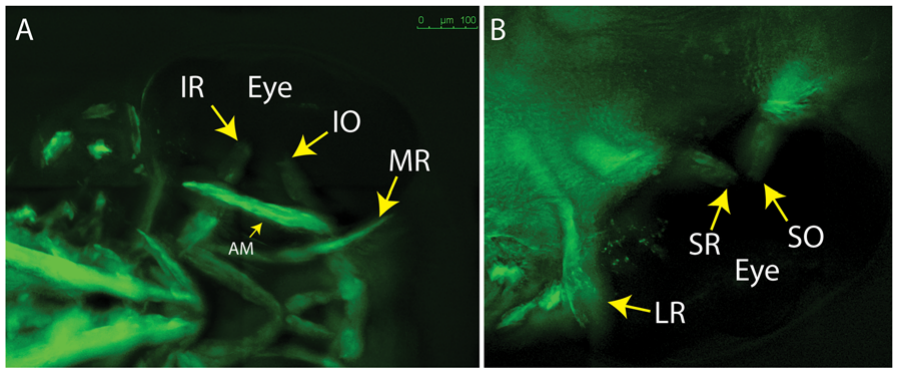
5 dpf embryos showing lack of overlap for SR/SO and IR/IO at their insertion sites (arrows). (A) ventral; (B) dorsal. Images reflect maximum projections following deconvolution of macroscopic Z-stacks. IR  =  inferior rectus; IO  =  inferior oblique; MR  =  medial rectus; AM  =  adductor mandibulae (jaw muscle – thin arrow); LR  =  lateral rectus; SR  =  superior rectus; SO  =  superior oblique.

**Figure 4:**
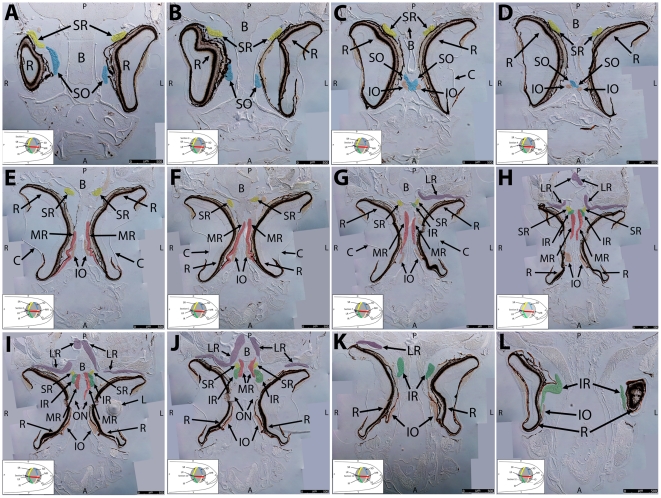
Key anatomical features within the WT adult zebrafish orbit are highlighted on 12 µ m thick coronal sections originally imaged at 200X magnification with DIC prisms to reveal topographical tissue architecture. Sections proceed in the dorsal (4A) to ventral (4L) direction and show all 6 muscles extending from origin to globe insertion. Please refer to the text for further details. Specific EOMs can be observed on the following figures: Superior oblique (4A–4D). Superior rectus (4A–4J). Inferior Oblique (4C–4L). Inferior rectus (4G–4L). Medial rectus (4E–4J). Lateral rectus (4G–4K). Anterior (A), posterior (P), left (L), and right (R) directions are noted on each frame and a schematic illustrating the specific plane of section is located in the lower left corner. Key features are labeled as follows: B – brain, C – cornea, IO – inferior oblique, IR – inferior rectus, LR – lateral rectus, L – lens, MR – medial rectus, ON – optic nerve, SO – superior oblique, SR – superior rectus, R – retina. For larger versions of the images, please refer to supplemental figures 1A-1L.

To image adult EOMs *in vivo*, adult *Tg(*α *-actin::EGFP)* zebrafish, ages 6 months and older, were anesthetized and evaluated using epifluorescent microscopy. Attention was first directed to the muscle insertions on the globe. Caudal rotation of the eye to expose the antero-medial orbit revealed that the medial rectus (MR) runs parallel to the long axis of the fish and inserts on the anterior side of the eye at the sclera-corneal (SC) junction (Figure 2A). The lateral rectus (LR) lies within the same anatomical plane as the MR, attached to its insertion site on the caudal side of the eye at the SC junction and extending deep into the orbit and out of view (Figure 2B). The dorsal side of the adult zebrafish eye revealed a significant overlap of the superior rectus (SR) and superior oblique (SO; Figure 2C) at their common insertion site near the SC junction (the figure is representative of at least 100 fish imaged in a similar fashion). This overlapping of insertions on the globe contrasted with embryo EOMs in which the oblique and rectus muscle insertions do not cross (Figure 3, representative of at least 200 embryos imaged). A similar overlap of the inferior oblique (IO) and inferior rectus (IR) muscles appeared to take place on the ventral side of the globe (Figure 2D), but these muscles were not as easily visualized *in vivo* because it required rotating the eye to the point of stretching the muscles. Further analysis of tissue sections confirms the overlap of the IO and IR at their insertions on the globe (Figure 4L). *In vivo* imaging clearly shows that the SO travels from a rostral origin point while the SR shows a path originating from a more caudal point. Hence, *in vivo* imaging of this transgenic strain provides an accurate assessment of zebrafish EOM anatomy.

In order to assess embryo and larval EOM anatomy, we evaluated the same strain microscopically *in vivo* using 5-day-old larvae (Figure 3). Interestingly, the SO and SR have distinct insertions onto the globe (Figure 3A,B), as do the IO and IR (Figure 3C). With over 200 embryos imaged, overlapping insertions were never noted. Therefore, the overlapping insertions develop later, as the larvae grow and reach adulthood. The significance of this is unclear.

### Two Dimensional Orbital Anatomy

**Coronal/Longitudinal Sections.** Whole head serial sections (12 µm thickness), obtained from 6 month old WT zebrafish, were evaluated for the presence of key anatomical landmarks and relationships. Images of anatomically descriptive sections were captured using differential interference contrast (DIC) microscopy**.** Throughout Figures 4A, B, C, D, E, F, G, H, I, J, K,L the plane of section is slightly oblique to the longitudinal axis and tilted slightly to the left. The left eye in each section has a slightly more ventral section depth compared to the right eye. The ordered sections proceed from dorsal (Figure 4A) to ventral (Figure 4L).

The SO and SR muscles are positioned close to each other near their overlapping insertion site on the dorsal side of the eye (Figure 4A). They deviate from each other as sections proceed ventrally and the muscles travel rostrally (SO) and caudally (SR) to their respective origin points (Figure 4B). Deeper sections reveal the common origin point of all four oblique muscles at the anterior ethmoid plate. The SO and IO muscles to both the left and right eye appear to meet at a central bony prominence where oblique EOM fibers overlap with each other (Figures 4C and D).

The MR muscle is displayed in longitudinal cross section as it passes immediately lateral to the IO muscle and inserts on the anterior side of the eye (Figure 4E). It can be seen along its pathway to the common origin point of the SR, MR, and IR as sections proceed in the caudal/ventral direction (Figure 4F). Eventually the MR originates from a midline bony plate where it is positioned just medial to the IR and SR (Figures 4G, H, I,J).

The SR and IR muscles overlap significantly as they meet and cross at their origins just lateral to the MR and attached to the same bony plate (Figures 4G, H, I,J). The shared origin and close proximity of the MR, IR, and SR may have functional consequences (see discussion).

The LR muscles cut in longitudinal cross-section travel from an origin point immediately inferior and posterior to the diencephalon, significantly caudal to the origin of the other rectus muscles (Figures 4I, 4J). The LR muscles deviate from each other as they travel rostrally and make a near 90-degree lateral turn before they pass lateral to the SR, MR, and IR origin and insert on the posterior side of the eye. The left and right LR are in very close proximity at their points of origin (Figures 4H and 4I) with significant crossing and overlapping of muscle fibers.

The IO and IR muscles travel in a similar pattern to the SO and SR muscles and eventually insert and cross with each other on the ventral side of the eye (Figure 4L). The IO muscles originate from the same central bony prominence as their paired SO muscles (Figures 4C and 4D). The IR muscle originates from the same bony plate as the MR and SR just lateral to the medial rectus origin (Figures 4G, H, I, J).

This slide set reveals that adult zebrafish possess conserved distinct origin sites for specific EOMs.

1. All four oblique muscles (2 SO and 2 IO) originate from a singular bony prominence positioned anterior to the other 4 muscles and equidistant from the left and right eye.
2. Both the left and right LR muscles originate from a central point immediately posterior to the diencephalon.
3. The paired MR, IR, and SR are respectively positioned medial to lateral at their central origin point. The left and right eye muscle group origins appear not to overlap significantly with each other.

High magnification inspection of Figure 4 mosaics reveals a distribution of myofiber diameter: at the origin and insertion points, fiber diameter ranges from 3 to 5 microns, while in mid-muscle, fiber diameters as large as 50–60 microns can be easily observed. This is nicely illustrated by SR and IO cross-sections in Figure 4E, F, G, H, I, J, K. The larger diameter myofibers of the SO, SR, IO, and IR appear to localize to the globe side of each muscle. For larger versions of the images, please refer to Figures S1, S2, S3, S4, S5, S6, S7, S8, S9, S10, S11, S12.

#### Transverse (Frontal) Sections

**Figure 5:**
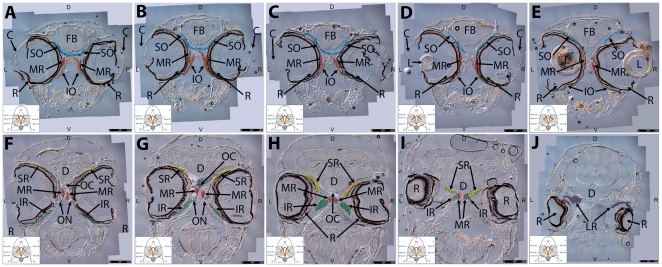
Key anatomical features within the WT adult zebrafish orbit are highlighted on 12 µ m thick transverse sections originally imaged at 200X magnification with DIC prisms to reveal topographical tissue architecture. Sections proceed in the rostral (5A) to caudal (5J) direction and show all 6 muscles extending from origin to globe insertion. Please refer to the text for further details. Specific EOMs can be observed on the following figures. Superior and inferior oblique (5A–5E). Superior and inferior rectus (5F–5I). Medial rectus (5A–5I). Dorsal (D), ventral (V), left (L), and right (R) directions are noted on each frame and a schematic illustrating the specific plane of section is located in the lower left corner. Key features are labeled as follows: C – cornea, D – diencephalon, FB – forebrain, IO – inferior oblique, IR – inferior rectus, LR – lateral rectus, L – lens, MR – medial rectus, OC – optic chiasm, ON – optic nerve, SO – superior oblique, SR – superior rectus, R – retina. For larger versions of the images, please refer to supplemental figures 2A-1J.

Using the same methods as for the coronal/longitudinal section, we evaluated transverse sections. Throughout figures 5A, B, C, D, E, F, G, H, I, J, the plane of section is approximately transverse and the ordered sections proceed rostral (5A) to caudal (5J).

The common origin point of the four oblique muscles (one inferior and one superior to each eye) is the most rostral (anterior) anatomical position of the zebrafish EOM anatomy. The four muscles can be seen medial to the MR, originating from a central bony prominence, and eventually inserting on the dorsal and ventral surfaces of the eye as sections proceed more caudally (Figures 5A,B, C, D, E).

Sections proceeding in the caudal direction reveal the SR and IR near their dorsal and ventral scleral insertion sites (Figure 5F). The MR continues to appear in cross section as it courses to its origin point shared with the SR and IR (Figure 5G). The MR, IR, and SR are respectively positioned medial to lateral at their shared origin (Figures 5H and 5I). The MR maintains an inferomedial position to the optic nerves as they travel superiorly toward the optic tectum (Figures 5G and 5H).

The most posterior sections of the eye display the LR as wide fans of muscle fibers running parallel to the plane of section at their globe insertion sites (Figure 5J). High magnification inspection of Figure 5 mosaics reveals a gradual progression from small to large myofiber diameter within the MR and LR (also observed in SR, SO, IR, and IO – see coronal mosaics). Myofiber diameter increases gradually from the superior to the inferior side of both the MR (5E) and LR (5J). For larger versions of the images, please refer to Figures S13, S14, S15, S16, S17, S18, S19, S20, S21, S22.

### Laminin Distribution via Immunohistochemistry

**Figure 6:**
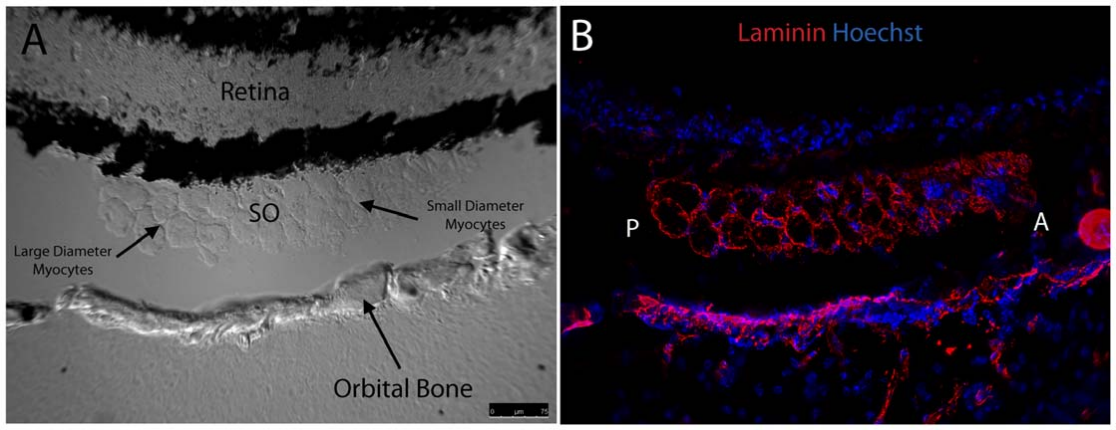
Fluorescent immunohistochemistry reveals uniform distribution of laminin throughout mid-muscle myocyte basement membranes. DIC imaging shows SO cut in mid-muscle cross section sandwiched between the globe and orbital bone with myocyte diameter increasing in the posterior to anterior direction (A). Laminin (red) is evenly distributed within basement membranes of individual myocytes throughout the entire muscle in cross section (B). Peripherally located nuclei, characteristic of muscle, are highlighted in blue. The anterior (A) and posterior (P) directions are noted in Frame B. Images were captured with a 20x objective lens. Red = Laminin; Blue = Nuclei.

**Figure 7:**
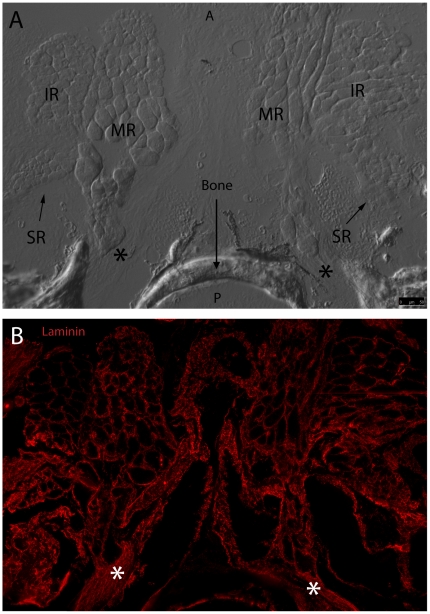
Fluorescent immunohistochemistry highlights laminin localization at the MR, SR, and IR origin site. DIC imaging reveals MR, SR, and IR from the left and right eye meeting at two common origin points with EOM tendons marked by asterisk (A). Fluorescent overlay shows laminin (red) evenly distributed throughout basement membranes of individual myocytes from all six muscles. Laminin appears to distribute evenly throughout EOM tendon and along the edge of bone as well (B). Anterior (A) and posterior (P) directions are noted (Frame A). Image tiles were captured with a 40x objective lens and merged into mosaics.

**Figure 8:**
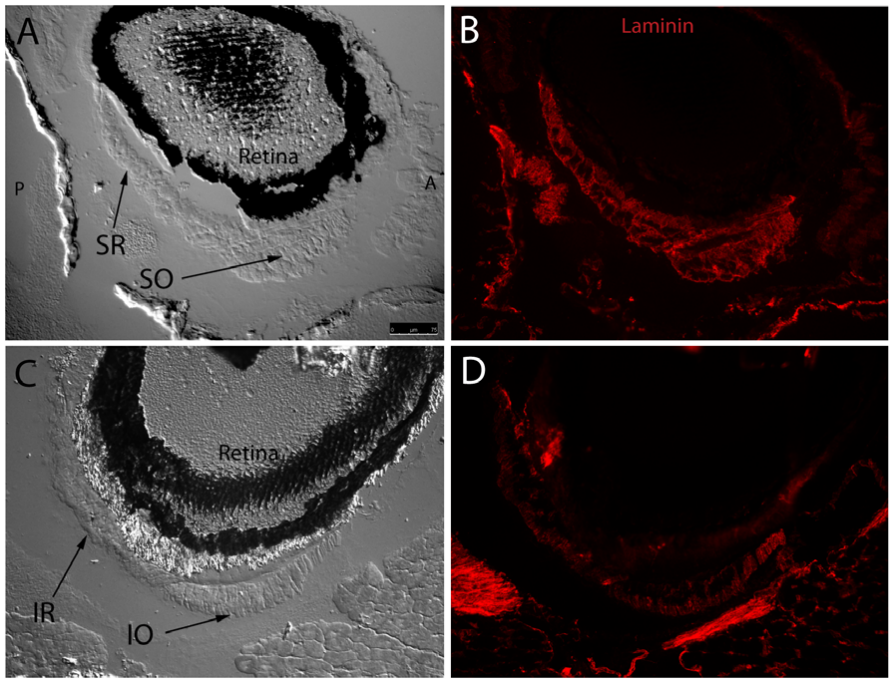
Fluorescent immunohistochemistry reveals an even distribution of laminin throughout myocyte basement membranes at the SR/SO and IR/IO insertion sites. DIC images show SR and SO overlapping near their globe insertion site on the superior side of the eye and IR and IO in a similar arrangement on the inferior side of the eye (A,C). Fluorescent overlays highlight uniform laminin (red) distribution throughout basement membranes of individual myocytes (B,D). Broader areas of laminin expression surrounding the muscle represent either broad laminin expression throughout a myotendinous junction or simply myocyte basement membrane cut in perfectly tangential section. Images were captured with a 20x objective lens.

Laminin is a basement membrane glycoprotein that is important for muscle function [18], [19]. Evaluation of laminin expression in zebrafish EOMs revealed even distribution throughout the basement membranes in all 6 EOMs. Expression level did not appear to vary between large and small diameter myofibers (Figure 6), and allowed for measuring fiber diameters across multiple muscles. These measurements revealed that small fiber diameters are in the 3–5 micron range, while large fiber diameters are in the 50–60 micron range. Each muscle contained interdigitating myofibers of different sizes. Additionally, laminin was localized to the outer edge of bone, and was broadly expressed in cartilage and EOM tendon at muscle origin sites (Figure 7) as well as the scleral insertion sites (Figure 8)

### Thrombospondin-4 distribution via immunohistochemistry

**Figure 9:**
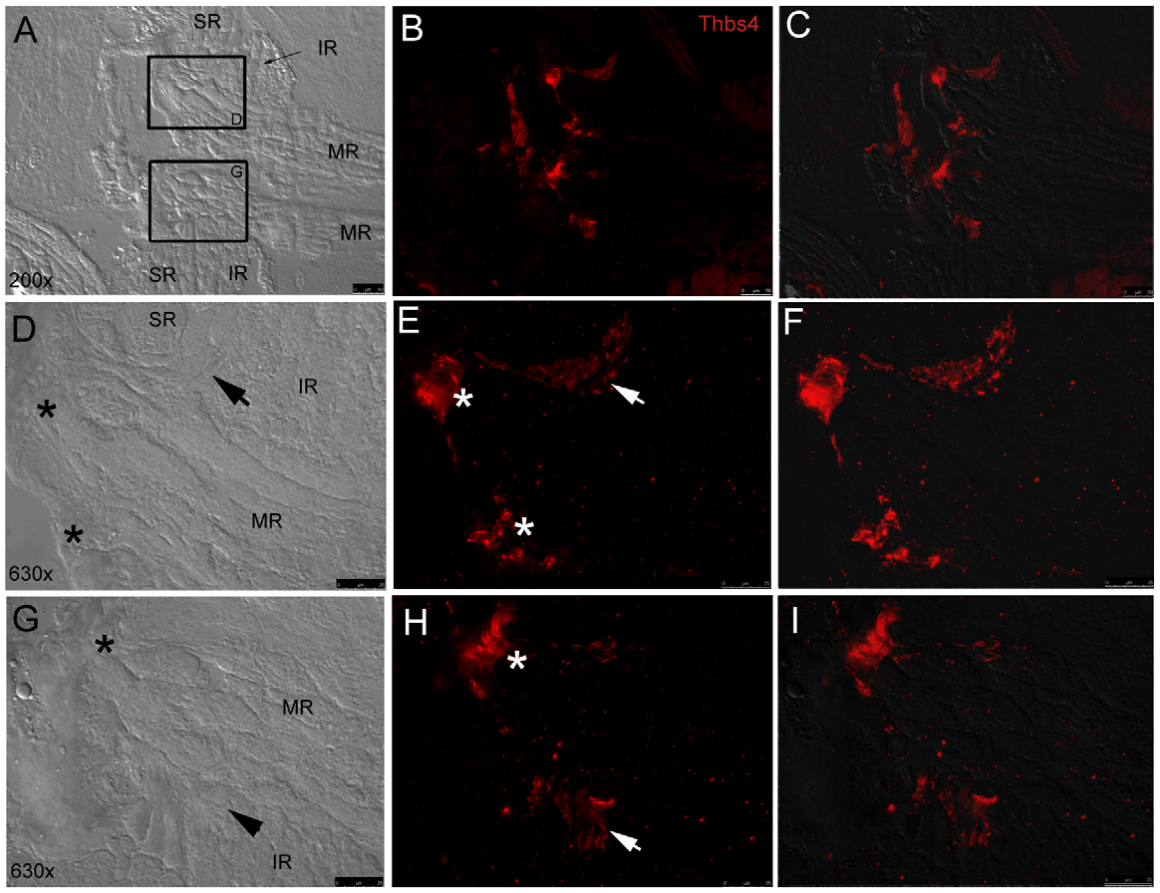
Fluorescent immunohistochemistry shows thbs-4 enrichment in EOM origin tendons. Low magnification (20x objective) DIC image (A) displays pairs of MR, SR, and IR converging at their common origin points with fluorescent thbs-4 (red) overlay (B) and fluorescent/DIC merged image (C). High magnification (63x) DIC images (D,G) show SR, IR, and MR attaching to bone with MR tendons (*) and IR tendons (arrows) marked. IR tendons marked with thbs-4 (E,F,H,I) are observed sandwiched at the furthest proximal point of the IR bordered by the MR and SR on either side. Thbs-4 is expressed in MR tendons (*) at their most proximal point as they insert on bone at their origin.

**Figure 10:**
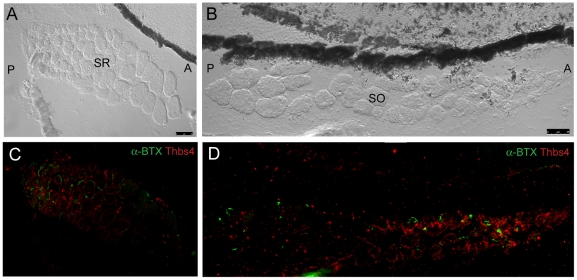
Fluorescent immunohistochemistry indicates that thrombospondin-4 (thbs-4) localizes to basement membranes of small diameter EOM myocytes. SR and SO are shown in transverse cross section (A,B – DIC). Thbs-4 (red) localizes to the basement membrane of smaller diameter myocytes and progressively decreases until becoming absent from large diameter myocyte basement membranes (C,D). Smaller diameter fibers appear to be more highly innervated as the density of α-BTX labeled NMJs (green) is greatest in the small diameter myocyte, thbs-4 expressing zone. Images were originally captured with a 40x objective lens and merged together to form mosaics.

Thbs-4 is an extracellular matrix glycoprotein that is associated with muscle tendon and promotes neurite growth [20], [21], [22]. Based on experimental observation of Thomas Schilling *et al.* (personal communication), we tested the hypothesis that EOM tendons are enriched with thbs-4. Immunolabelling confirmed that thbs-4 is a component of zebrafish EOM tendon at muscle origins (Figure 9). Very low levels of thbs-4 staining can also be observed at scleral insertion sites, in which the tendon is very attenuated, and consistent with Jaggi et al. who described human MR and LR insertions with minimal tendinous connective tissue [23]. Interestingly, Thbs-4 is enriched in basement membranes of the small diameter myofibers observed in SR and SO at mid-muscle in transverse sections (Figure 10).

### Neuromuscular Junction Distribution, Density, and Morphology

**Figure 11:**
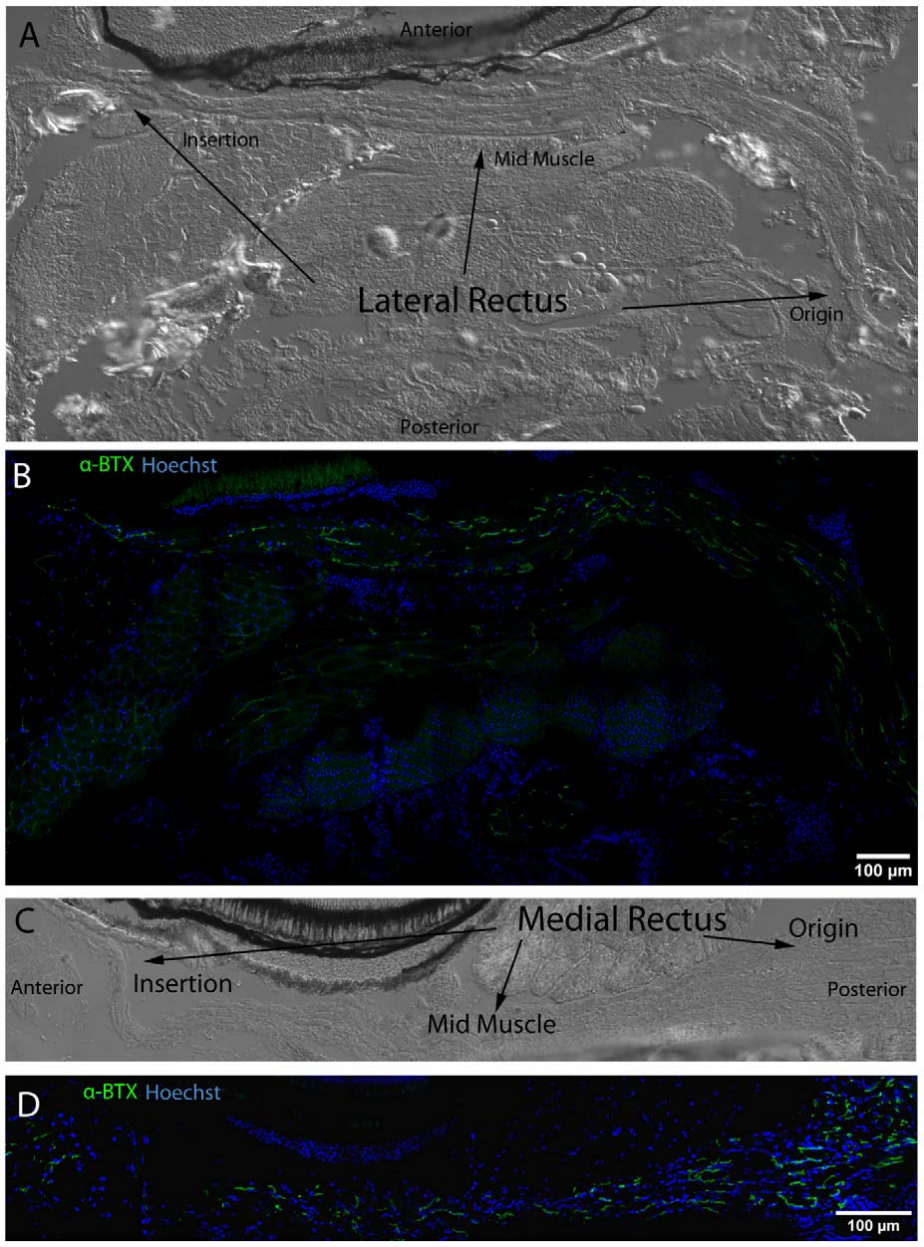
NMJ post-synapses labeled with α–BTX (green) reveal even distribution from EOM origin to insertion. DIC images display full length LR and MR in longitudinal section from origin to insertion (A,C). NMJs (green) labeled with fluorescently conjugated α-BTX are distributed throughout both muscles from origin to insertion and do not appear to be organized into any particular longitudinal pattern across EOM (C,D). Nuclei are stained blue with Hoechst nuclear stain throughout. Mosaics were originally captured using a 63x objective lens.

**Figure 12:**
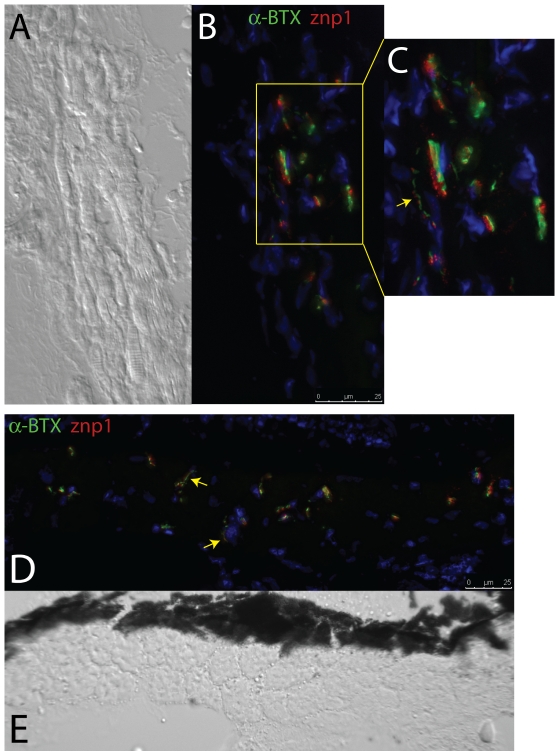
Zebrafish EOMs contain both *en plaque* and *en grappe* NMJs. (A) DIC and (B) immunofluorescence (IF) images of NMJs in longitudinally sectioned EOMs double stained with both pre- and post-synpatic markers (znp1 and α-BTX), revealing under high-magnification (C) the presence of both *en plaque* and *en grappe* (arrow) junctions, the former more numerous than the latter. Transverse sections (IF, D, and DIC, E) also reveal the presence of *en grappe* junctions (arrows), with palisading fibers. Red = Synaptotagmin/znp1; Green =  α-BTX.

In order to identify and study the morphology and distribution of NMJs, we used α-BTX which binds irreversibly to post-synaptic acetylcholine receptors, and anti-synaptotagmin antibodies (znp1) to mark presynaptic structures. NMJs appear to be distributed relatively evenly throughout the body of the MR and LR muscles (Figure 11), although imaging of the SR and SO near their tendons (identified by Thbs-4 Ab) suggests increased number of NMJs near tendons (Figure 10). Post-synaptic density was evaluated in each of the muscles by determining the combined surface area of α-BTX per mm^2^ of muscle tissue using ImageJ software. LR (.0324 mm^2^ NMJ/1 mm^2^ muscle) and MR (.0283 mm^2^/1 mm^2^ muscle) revealed a roughly 6 fold greater post-synaptic density compared to mid-body somitic muscle (.0061 mm^2^ NMJ/1 mm^2^ muscle). We chose to evaluate NMJ density using synapse surface area vs. total muscle surface area because the highly variable morphology of NMJs in EOMs introduces subjectivity when attempting to count individual junction points. Transverse cross sections of SR and SO indicate that synaptic density is greatest in the region of small diameter myofibers that are also enriched for the glycoprotein thbs-4, near the muscle tendons (Figure 10). Microscopic evaluation of the appearance of NMJ structures, using specimens that were double-immunolabeled with α-BTX (post-synaptic) and anti-synaptotagmin (pre-synaptic) antibodies, suggested the presence of both *en plaque* and *en grappe* synaptic junctions, although there was not a clear distribution pattern (Figure 12). NMJs were identified based on their anatomic appearance, with *en plaque* junctions visualized as broad synaptic junctions, and *en grappe* junctions visualized as “beads/grapes on a string” (arrows in Figure 12) [12], [13].

## Discussion

Comparing the gross and microscopic anatomy of zebrafish and human EOMs reveals broad anatomic and structural similarities along with some important differences. The similarities include the overall organization, *i.e.* 4 rectus and 2 oblique muscles that insert on the globe at prototypical locations commensurate with their function. Comparing EOM insertion on the globe between embryos and adults revealed that while the overall organization is stably formed by the larval stage, there are overlapping insertions of the SO and SR muscles, as well as the IO and IR muscles. This latter point may be important: in humans, the SO and SR muscle insertions overlap, and the IO path crosses over the IR before inserting posterolateral to the IR insertion point. Hence, the close anatomic relationships of the SO/SR and IO/IR pairs appear to have been evolutionarily conserved.

Furthermore, key components of EOM microanatomy are comparable between zebrafish and humans and indicate that there is significant structural similarity. Laminin is an important extracellular matrix and basement membrane protein that influences many biological processes including cell proliferation, adhesion [24], migration [25], NMJ formation [26], and even myocyte survival [27], [28]. Identical to what has been observed in human EOM [29], laminin localization in zebrafish EOM basement membrane is uniform throughout the entire length of the muscle, does not show preference for any cross sectional myofiber zone (e.g. OL vs. GL), and envelopes the myotendinous junctions. The even distribution of laminin in EOM basement membrane supports its use as a reliable marker for overall muscle anatomy and myofiber organization. Any observed changes in laminin expression levels or localization are likely to be due to actual disruptions in myofiber structure accompanying active disease or repair processes.

Microscopic evaluation of NMJ morphology in zebrafish EOMs revealed the presence of both the more common *en plaque* and the less common *en grappe* synapses, in agreement with mammalian EOM synapses. Assessment of NMJ morphology was based on appearance rather than molecular markers, and future evaluations of the NMJs in zebrafish EOMs may provide additional information. The distribution of NMJs was not uniform, with higher synaptic densities around muscle origins and in the context of smaller-diameter myofibers. The synaptic-density gradients we observed in EOM transverse cross sections likely explains the discrepancy with longitudinal sections. A more thorough analysis of synaptic density will require 3D-reconstruction of full thickness EOMs in order to account for zone specific density differences within individual muscles.

There are two notable gross anatomical differences between the zebrafsh and human orbit: zebrafish LR muscles originate substantially posterior to the other rectus muscles, and the zebrafish oblique muscles originate together from the anterior ethmoid plate. The SO does not pass through a trochlea as it does in humans. Interestingly, the near 90-degree lateral turn of the LR in the zebrafish allows this muscle to generate a primary tension vector originating from the general direction of the shared MR/SR/IR origin, just as LR does in humans. The human SO tension vector originates from a trochlea on the superomedial side of the orbit rather than from the muscle origin at the orbital apex. Considering the lack of a trochlea to modify the direction of action of the SO in the zebrafish orbit, it is fitting that this muscle does not originate with the MR, SR, and IR muscles as it does in humans. The zebrafish SO origin must be positioned separately from the rectus muscle origins in order to generate a tension vector similar in direction to that of the human SO.

Notably, the gross anatomic differences between zebrafish and human EOM organization generally cause little difference in the role that each muscle plays in the generation of eye movements. However, three of the four rectus muscles on each side share a caudal origin, and all the oblique muscles share a rostral origin. As these muscles extend toward the eye and insert on the globe, they maintain close anatomic relationships to one another. Interestingly, the muscles that have the longest areas of contact with one another are the ones that are innervated by the oculomotor nerve: MR, SR, IR and IO. The LR and SO muscles (innervated by the abducens and trochlear nerves, respectively) make less contact with the other EOMs. The functional significance of that is unclear, but evolutionarily this likely reflects a shared embryonic ancestry, in that the LR and SO originate from paraxial mesoderm, whereas the other 4 EOMs originate from prechordal head mesoderm [16].

The organization of human EOM into an outer orbital layer (OL) and an inner global layer (GL) is generally absent in zebrafish EOM. Transverse cross sections of all 6 zebrafish EOMs reveal that large-diameter myofibers are closer to the globe, while smaller-diameter myofibers are on the orbital side. However, this likely reflects continuously added myocytes at the origins and insertion, the result of the life-long growth that is characteristic of fish. Interestingly, both small and large diameter myofibers contact both the globe and the orbit in zebrafish. Mid muscle cross-section of zebrafish EOM reveals extremely thin muscles (when compared with human EOM) that are a mere 2–3 myocytes thick in the large diameter zone and 5–6 myocytes thick in the small diameter myofiber zone. The thickest myofibers of mature fish were found to measure only 50–60 microns in diameter. It is possible that the thin nature of zebrafish EOM precludes true division between orbital and global layering.

Thbs-4 is the first identified specific marker for zebrafish EOM tendons (Thomas Schilling, personal communication). This extracellular matrix glycoprotein is also as a component of small zebrafish EOM myocyte basement membrane but is absent from larger diameter myofibers. The progressive decrease in myocyte diameter across EOM cross sections, combined with the correlative increase in thbs-4 presence, suggests that thbs-4 may play a role in muscle growth. We also found that NMJ density is greatest in the small diameter myofibers that express thbs-4, which may reflect the reported role of thrombospondin in neurite development [20].

In summary, despite some important differences, Zebrafish EOMs generally exhibit significant similarity to human EOMs, extending from the level of gross anatomy down to the NMJ structures and the individual glycoprotein components of myocyte basement membranes. Zebrafish are widely accepted as a powerful model for the study of mechanisms driving disease processes and tissue repair. We conclude that the significant degree of anatomical homology between zebrafish and humans provides researchers with a powerful tool to study EOM disease and tissue repair mechanisms in an organism that is well suited to genetic manipulation.

## Supporting Information

Figure S1

Figure S2

Figure S3

Figure S4

Figure S5

Figure S6

Figure S7

Figure S8

Figure S9

Figure S10

Figure S11

Figure S12 **Key anatomical features within the WT adult zebrafish orbit are highlighted on 12** µ**m thick coronal sections originally imaged using 200X magnification with DIC prisms to show topographical tissue architecture.** Sections proceed in the dorsal (S1) to ventral (S12) direction and show all 6 muscles extending from origin to globe insertion. A detailed analysis of the anatomy can be found in the “Results” section of this paper. For easy reference, specific EOMs can be observed on the following figures. Superior oblique (S1–S4). Superior rectus (S1–S10). Inferior Oblique (S3–S12). Inferior rectus (S7–S12). Medial rectus (S5–S10). Lateral rectus (S7–S11). Anterior (A), posterior (P), left (L), and right (R) directions are noted on each frame and a schematic illustrating the specific plane of section is located in the lower left corner.
(TIF)

Figure S13

Figure S14

Figure S15

Figure S16

Figure S17

Figure S18

Figure S19

Figure S20

Figure S21

Figure S22 **Key anatomical features within the WT adult zebrafish orbit are highlighted on 12** µ**m thick transverse sections originally imaged using 200X magnification with DIC prisms to show topographical tissue architecture.** Sections proceed in the rostral (S13) to caudal (S22) direction and show all 6 muscles extending from origin to globe insertion. A detailed analysis of the anatomy can be found in the “Results” section of this paper. For quick reference, specific EOMs can be observed on the following figures. Superior and inferior oblique (S13–S17). Superior and inferior rectus (S18–S21). Medial rectus (S18–S21). Dorsal (D), ventral (V), left (L), and right (R) directions are noted on each frame and a schematic illustrating the specific plane of section is located in the lower left corner.
(TIF)
