# Supplementary figures and images for: Microanatomy of Adult Zebrafish Extraocular Muscles
Source: PLoS One. 2011 Nov 23;6(11):e27095. doi: 10.1371/journal.pone.0027095 (PMC3223174; doi:10.1371/journal.pone.0027095)

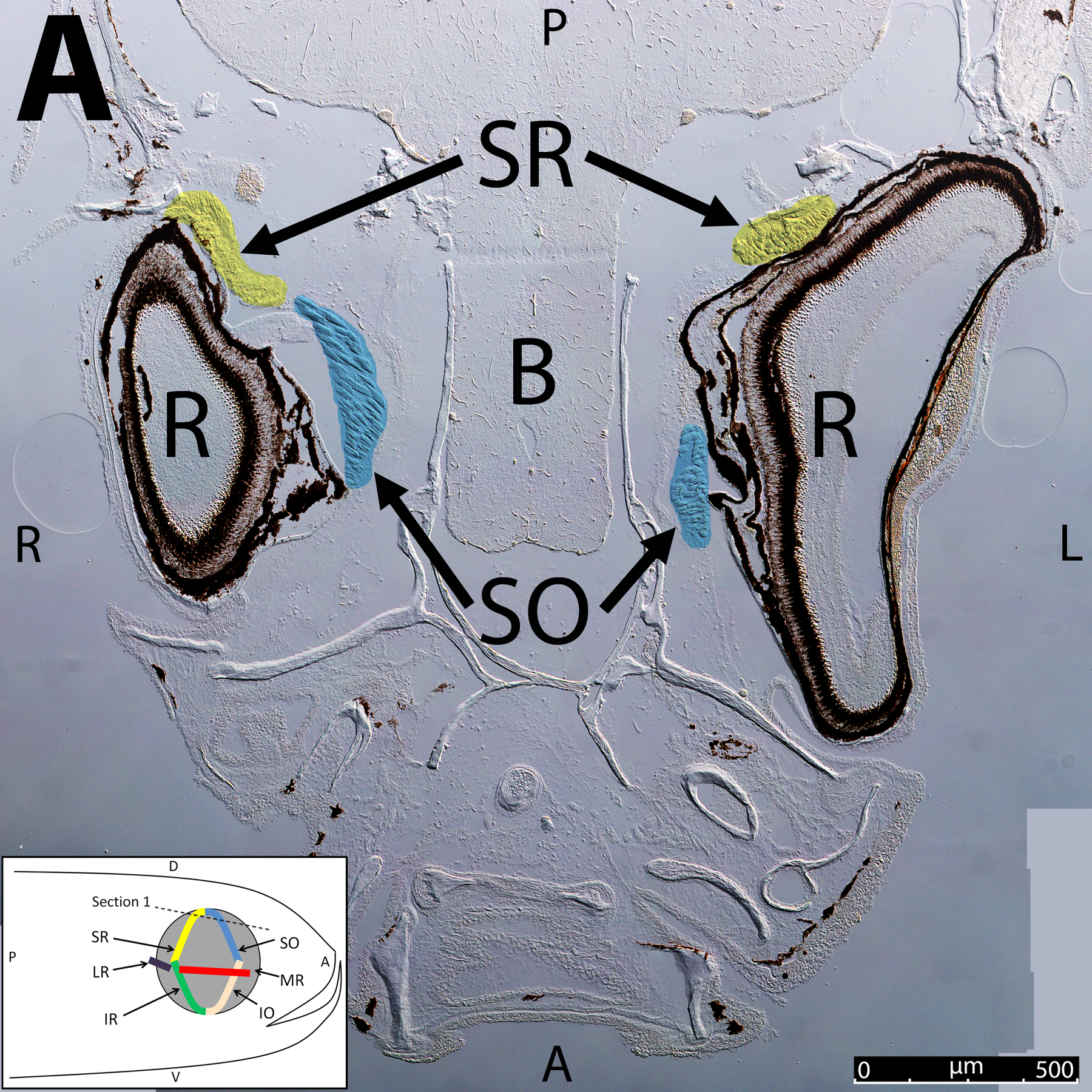

Supplement: Figure S1 [file pone.0027095.s001.tif]

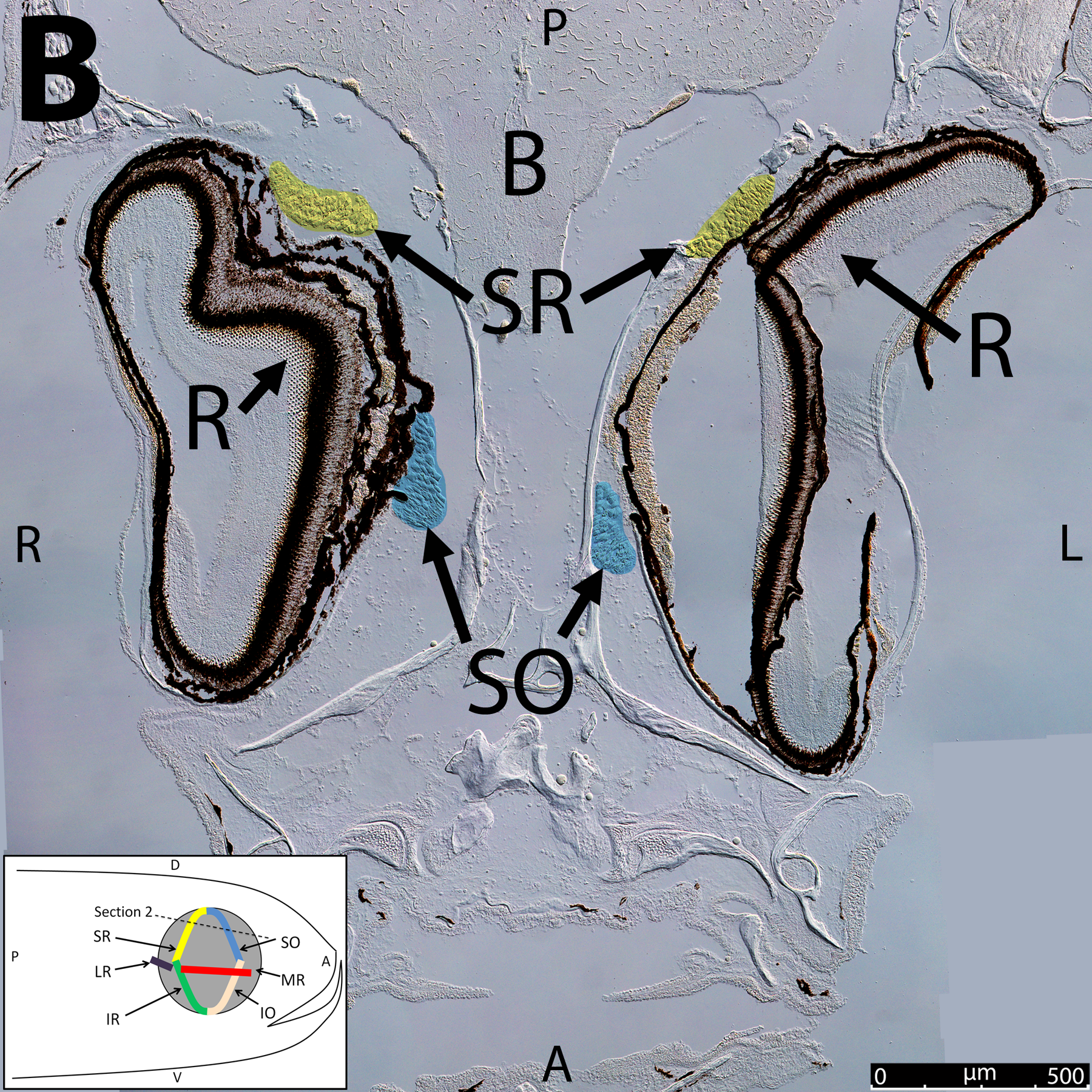

Supplement: Figure S2 [file pone.0027095.s002.tif]

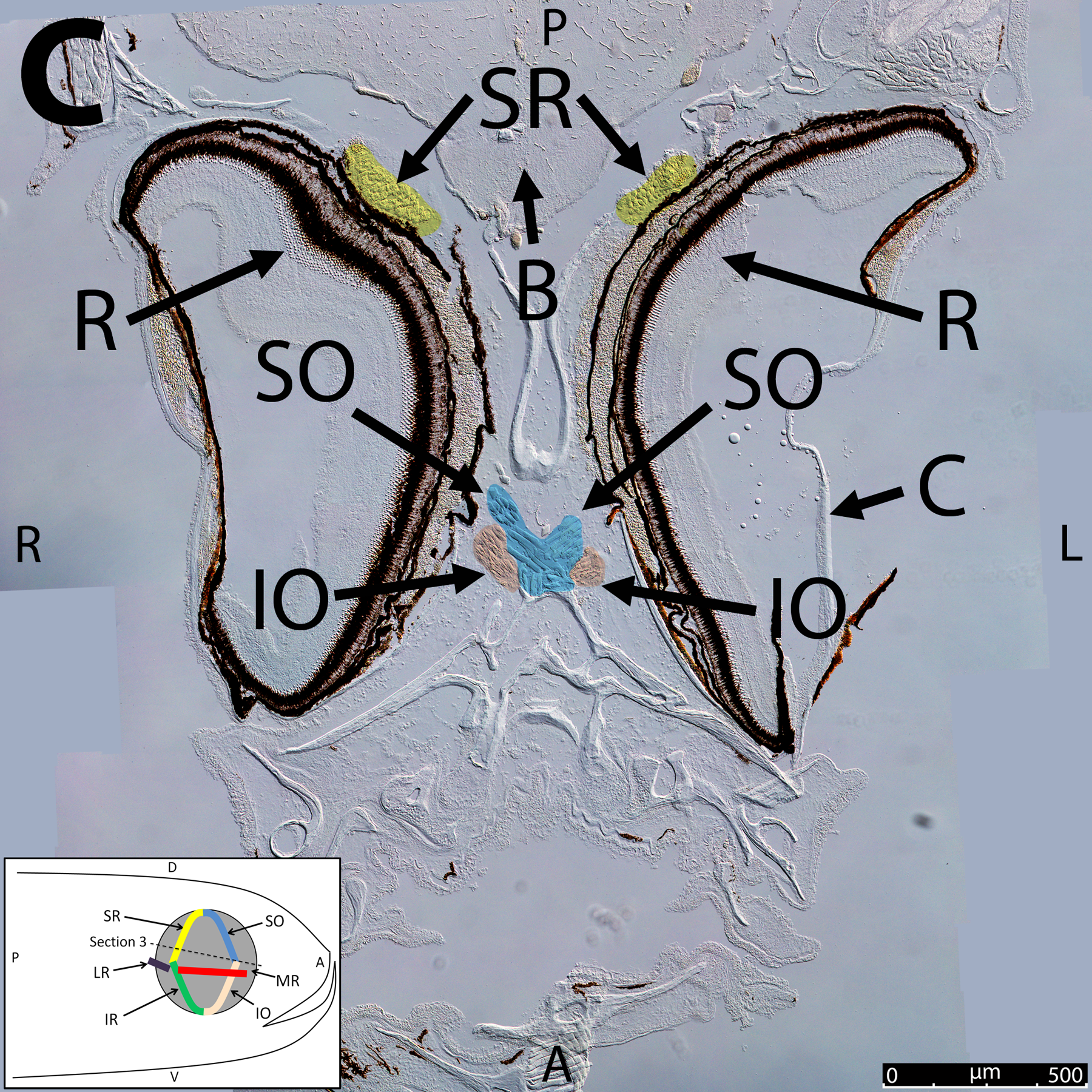

Supplement: Figure S3 [file pone.0027095.s003.tif]

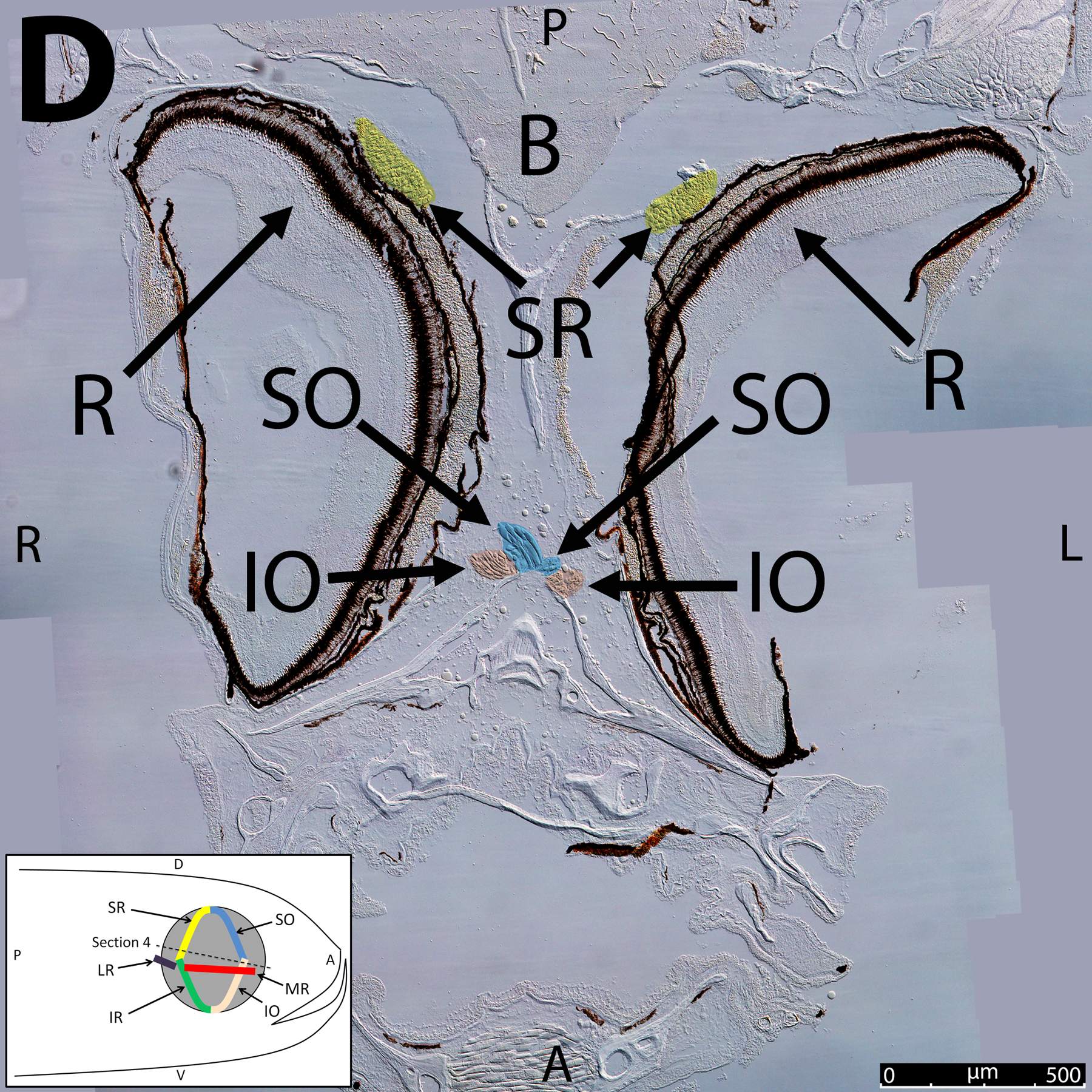

Supplement: Figure S4 [file pone.0027095.s004.tif]

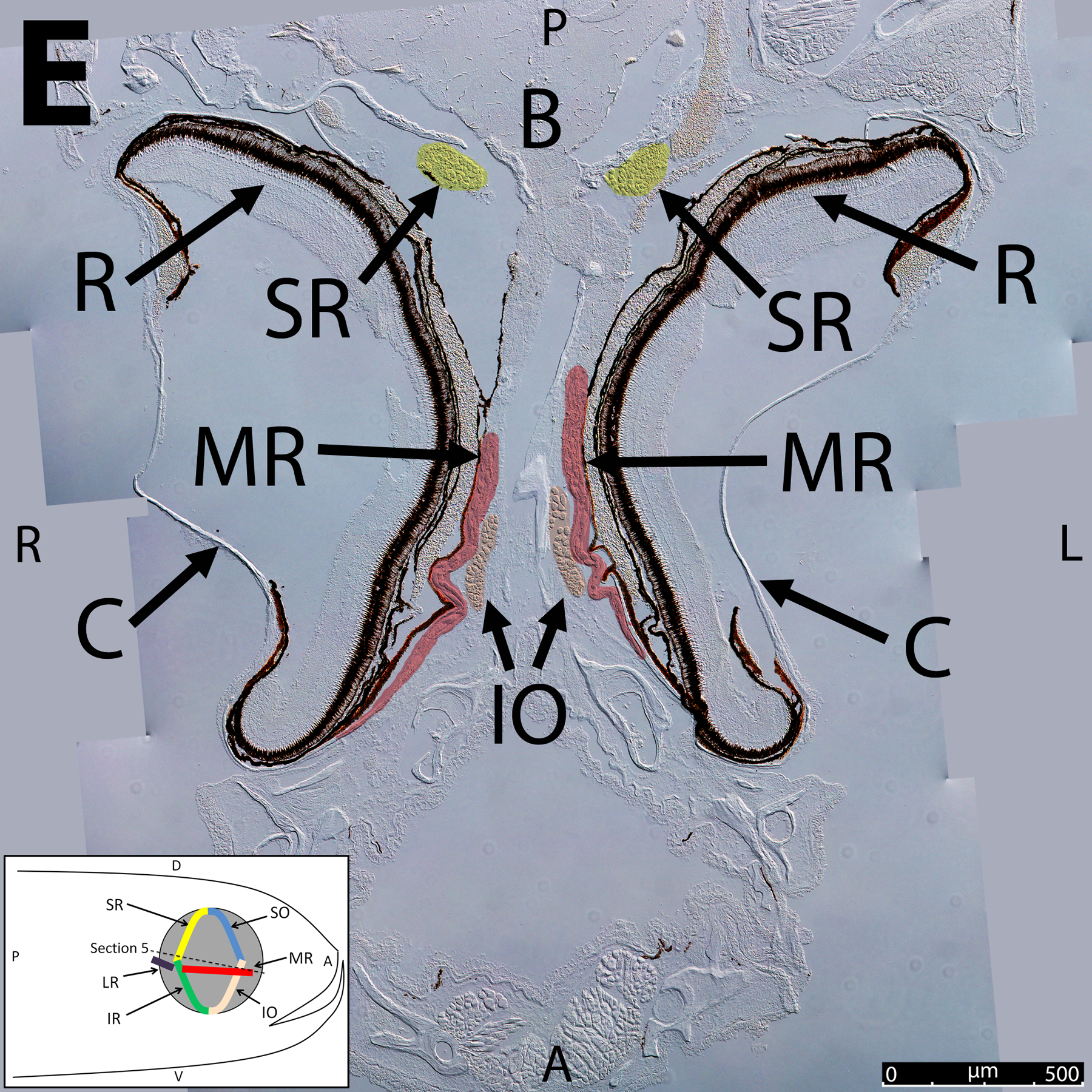

Supplement: Figure S5 [file pone.0027095.s005.tif]

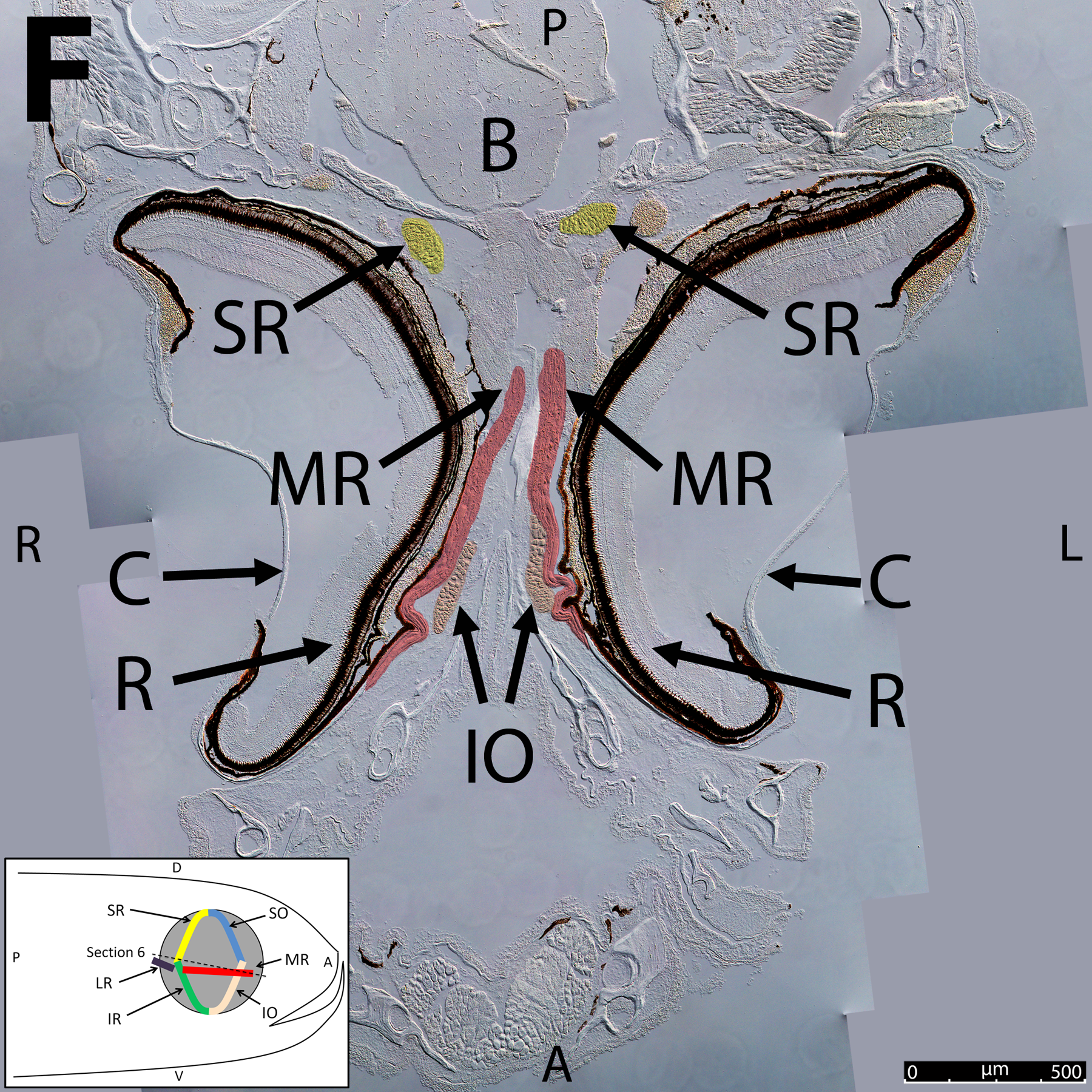

Supplement: Figure S6 [file pone.0027095.s006.tif]

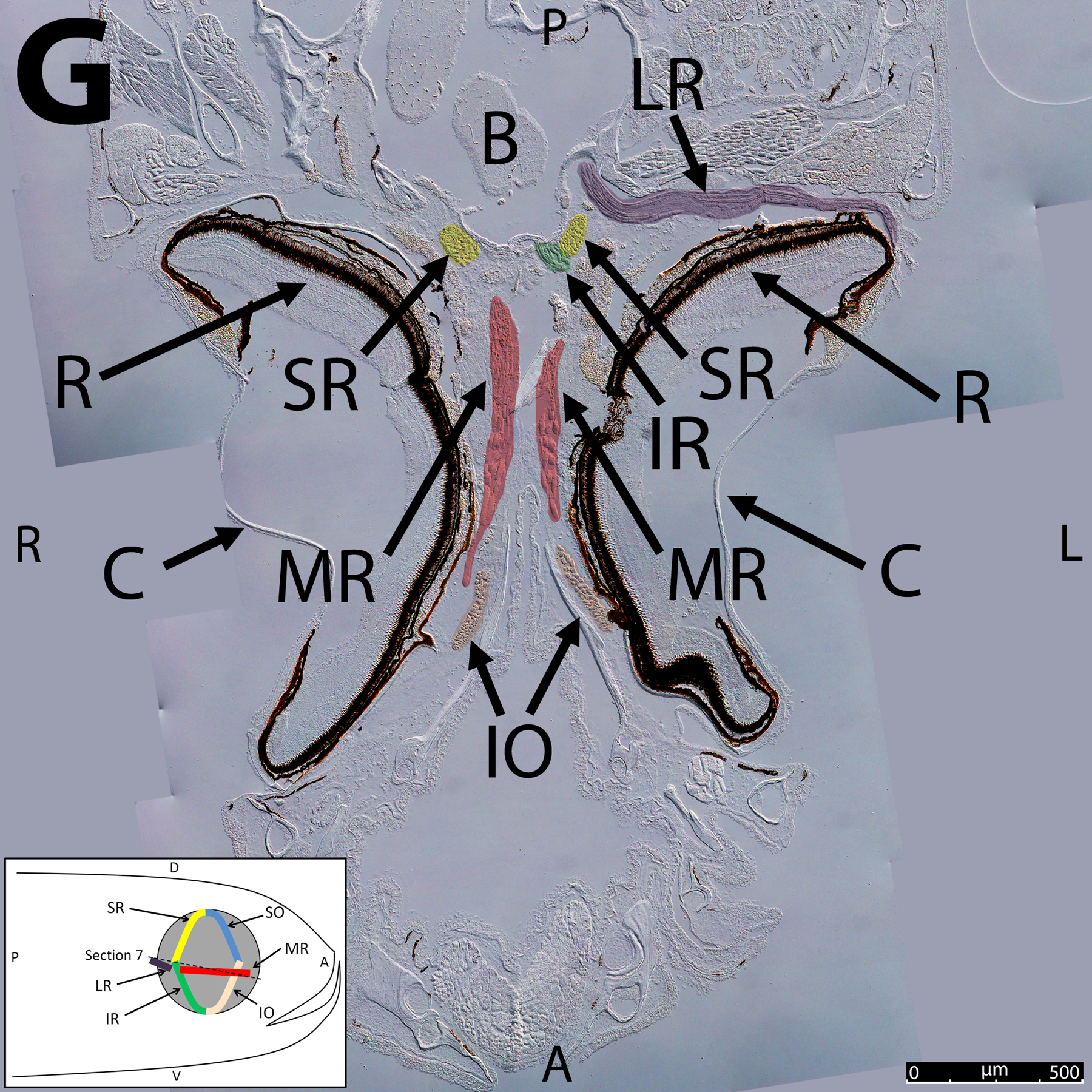

Supplement: Figure S7 [file pone.0027095.s007.tif]

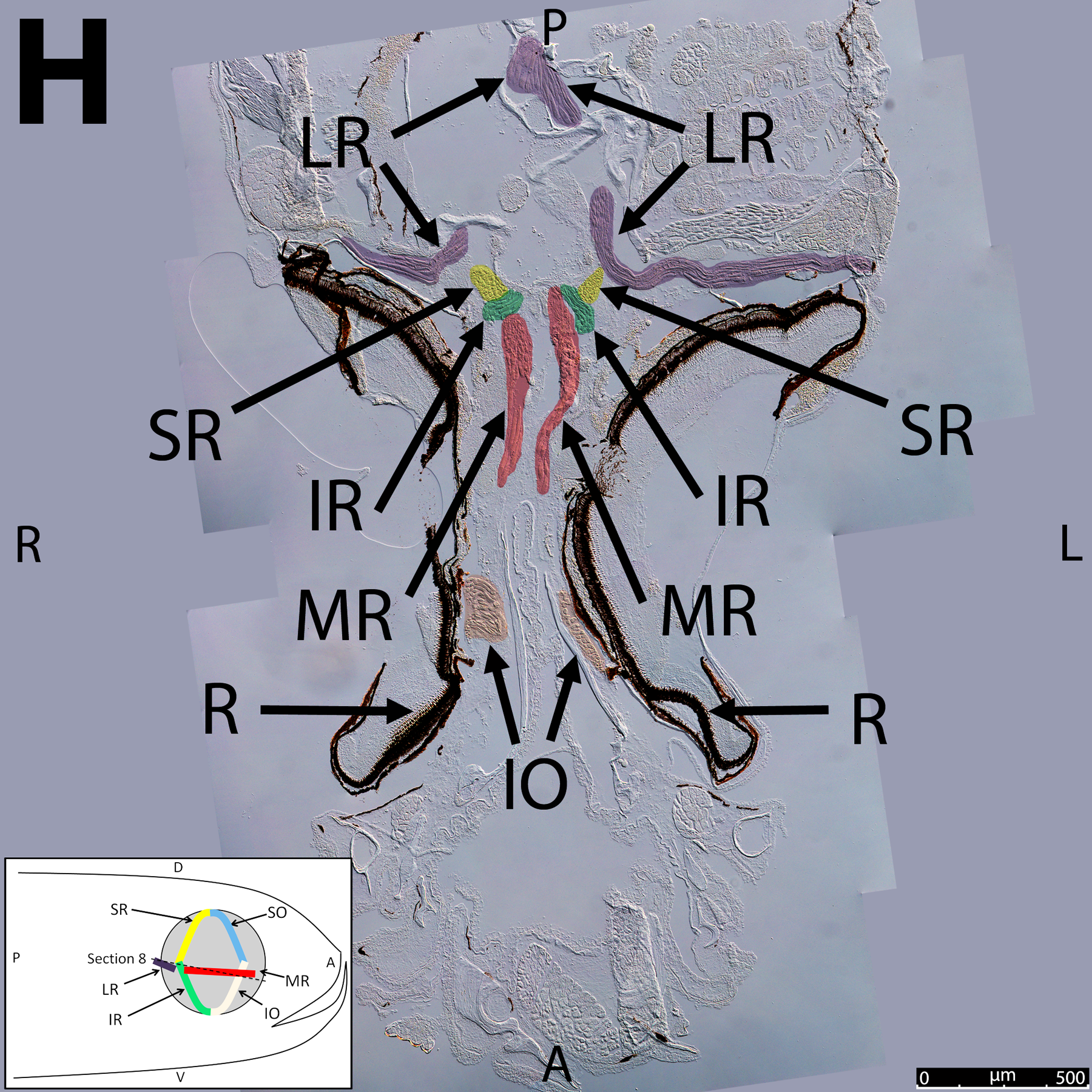

Supplement: Figure S8 [file pone.0027095.s008.tif]

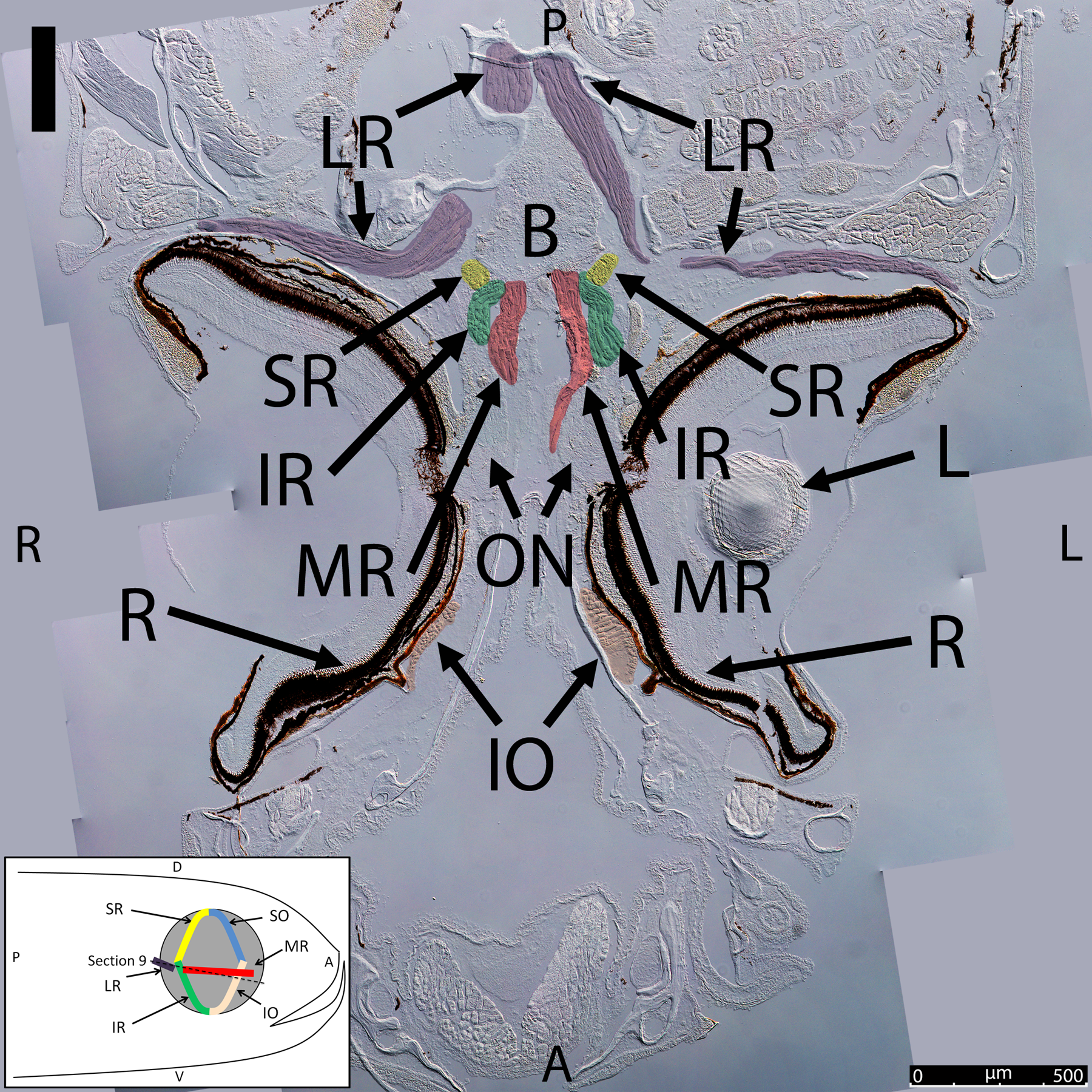

Supplement: Figure S9 [file pone.0027095.s009.tif]

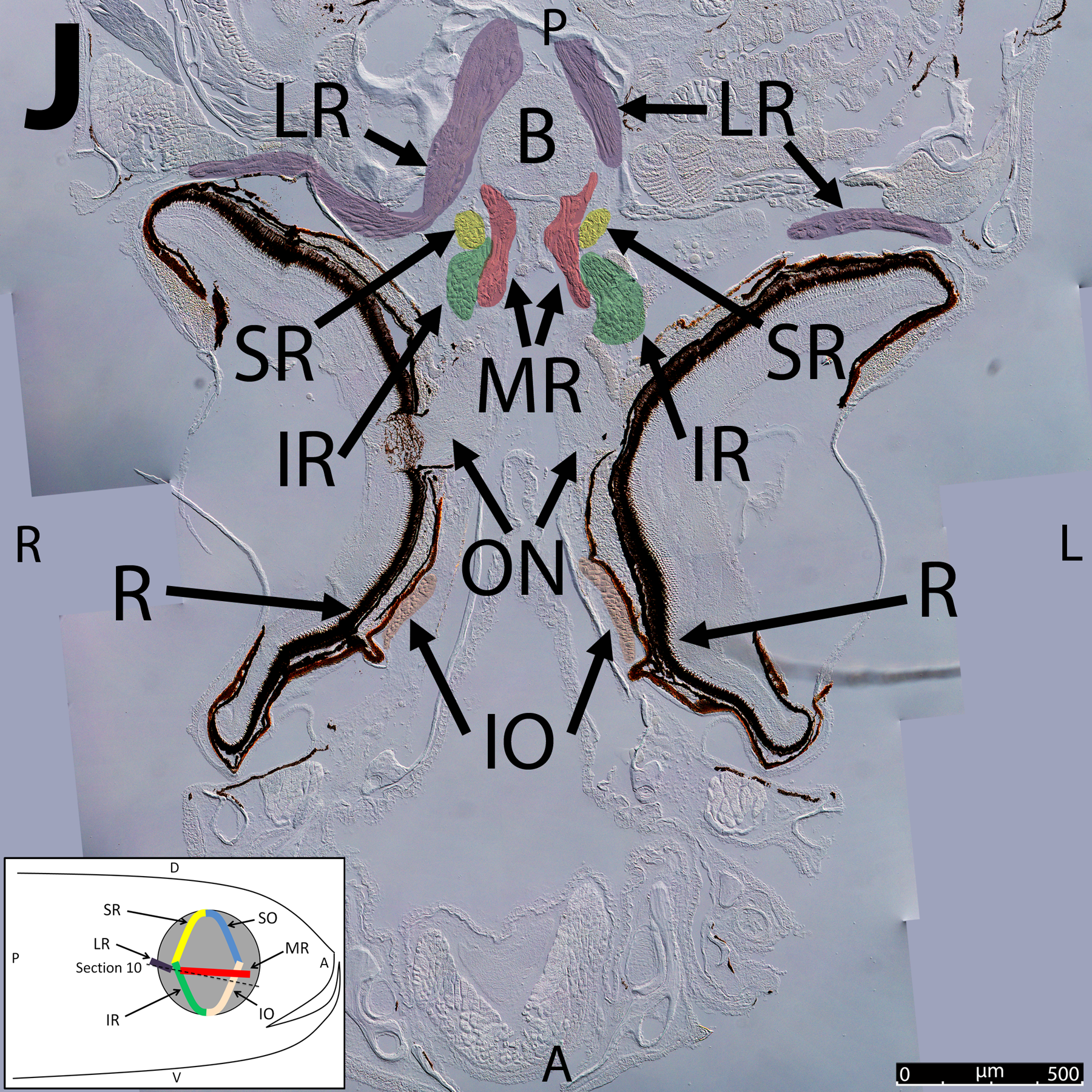

Supplement: Figure S10 [file pone.0027095.s010.tif]

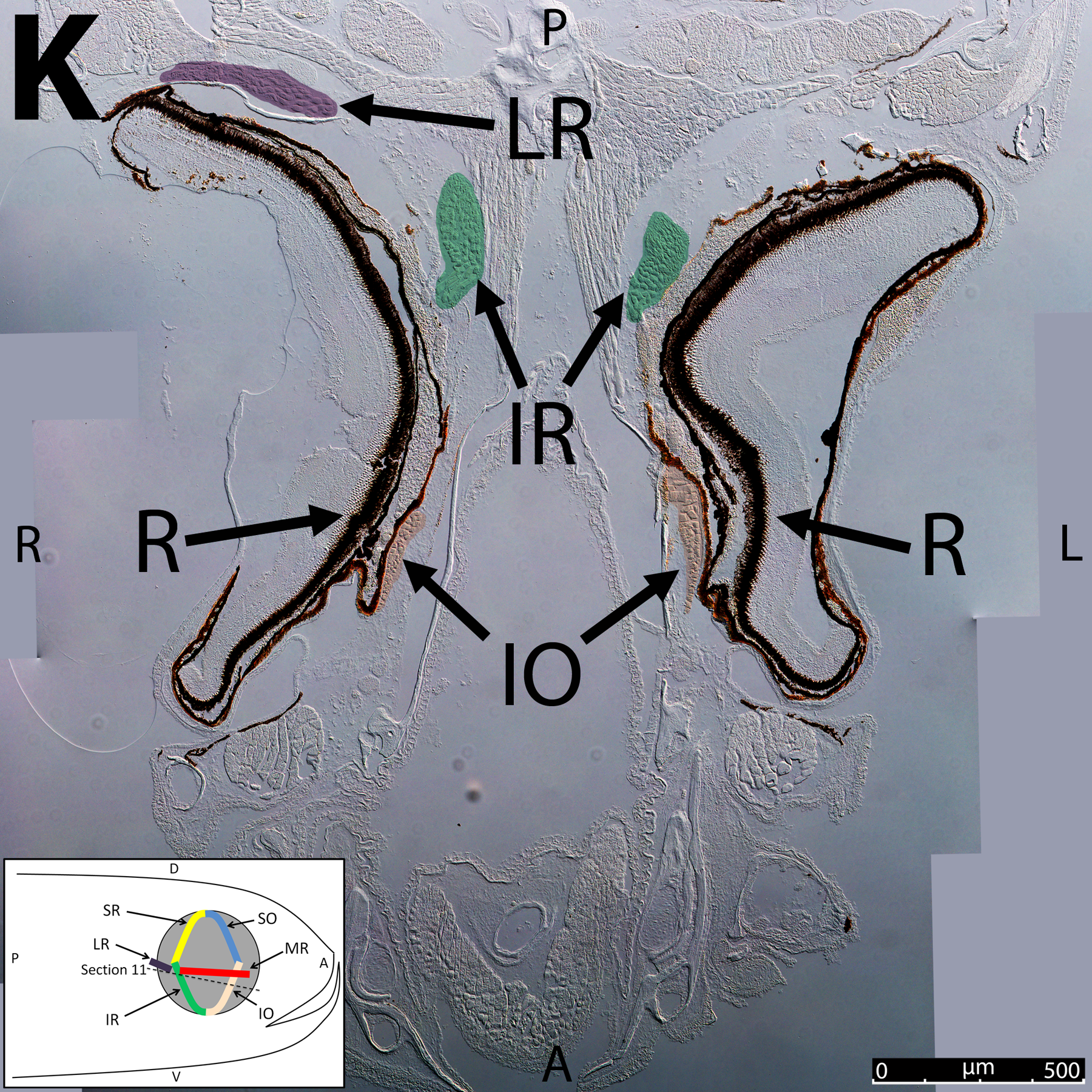

Supplement: Figure S11 [file pone.0027095.s011.tif]

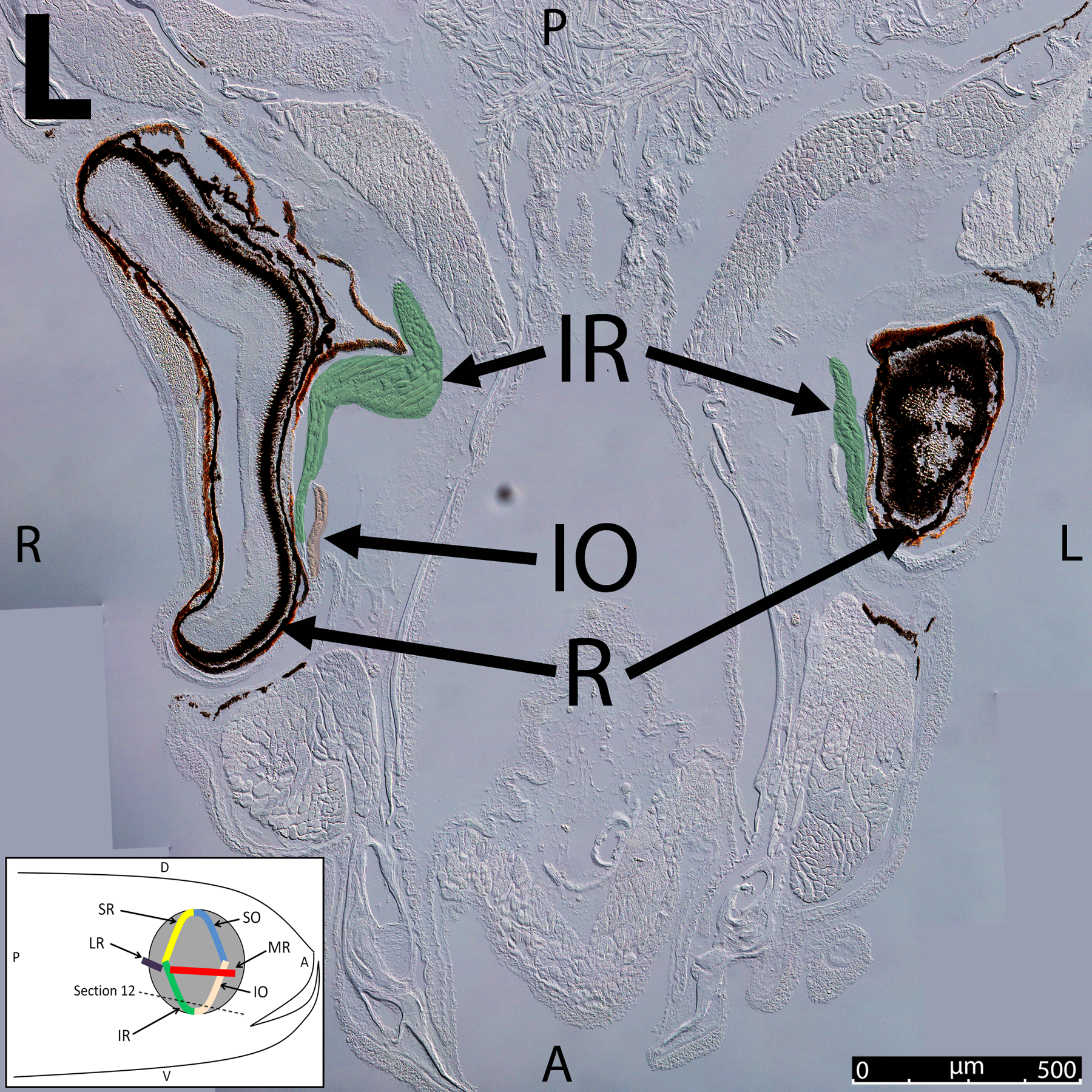

Supplement: Figure S12 — Key anatomical features within the WT adult zebrafish orbit are highlighted on 12 µm thick coronal sections originally imaged using 200X magnification with DIC prisms to show topographical tissue architecture. Sections proceed in the dorsal (S1) to ventral (S12) direction and show all 6 muscles extending from origin to globe insertion. A detailed analysis of the anatomy can be found in the “Results” section of this paper. For easy reference, specific EOMs can be observed on the following figures. Superior oblique (S1–S4). Superior rectus (S1–S10). Inferior Oblique (S3–S12). Inferior rectus (S7–S12). Medial rectus (S5–S10). Lateral rectus (S7–S11). Anterior (A), posterior (P), left (L), and right (R) directions are noted on each frame and a schematic illustrating the specific plane of section is located in the lower left corner. (TIF) [file pone.0027095.s012.tif]

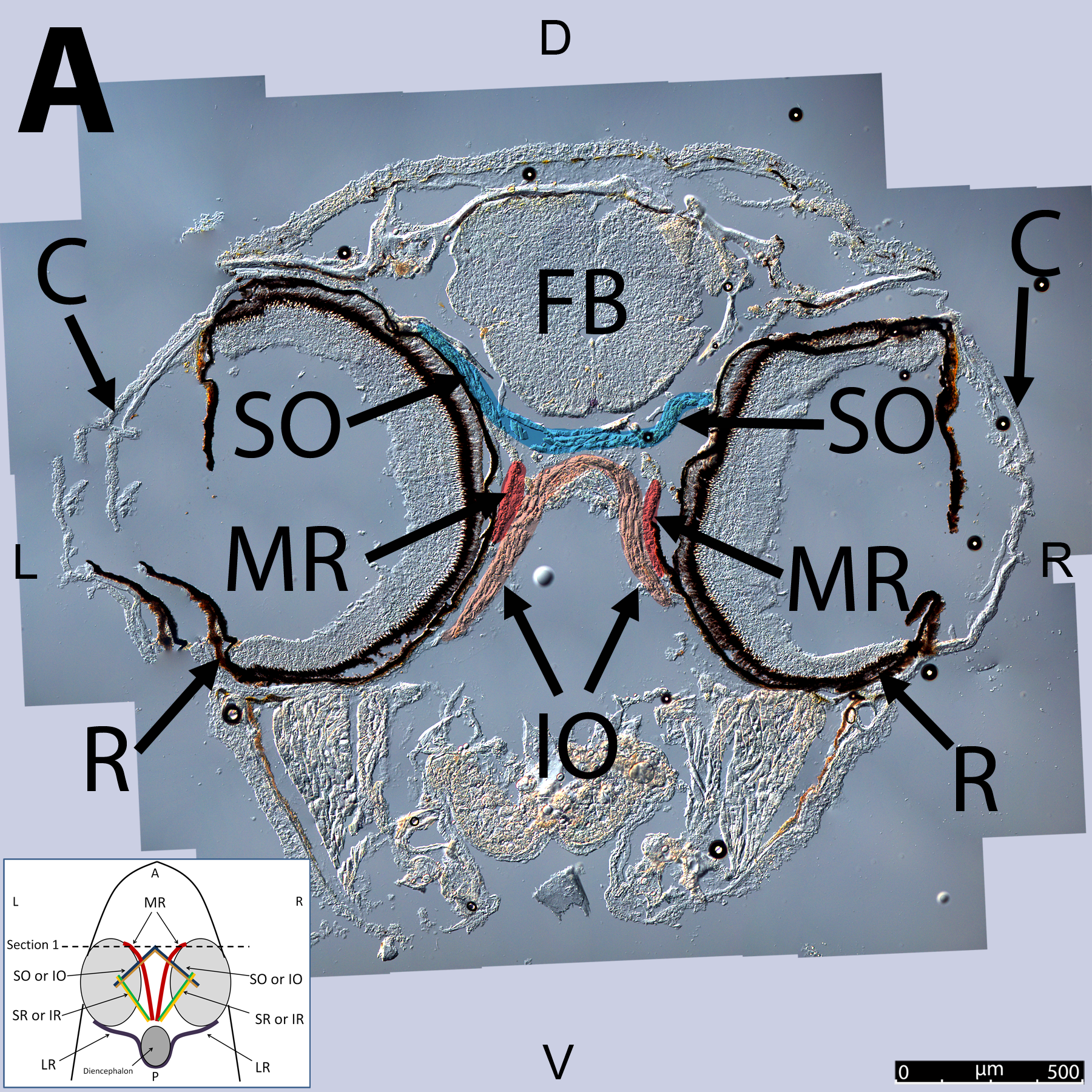

Supplement: Figure S13 [file pone.0027095.s013.tif]

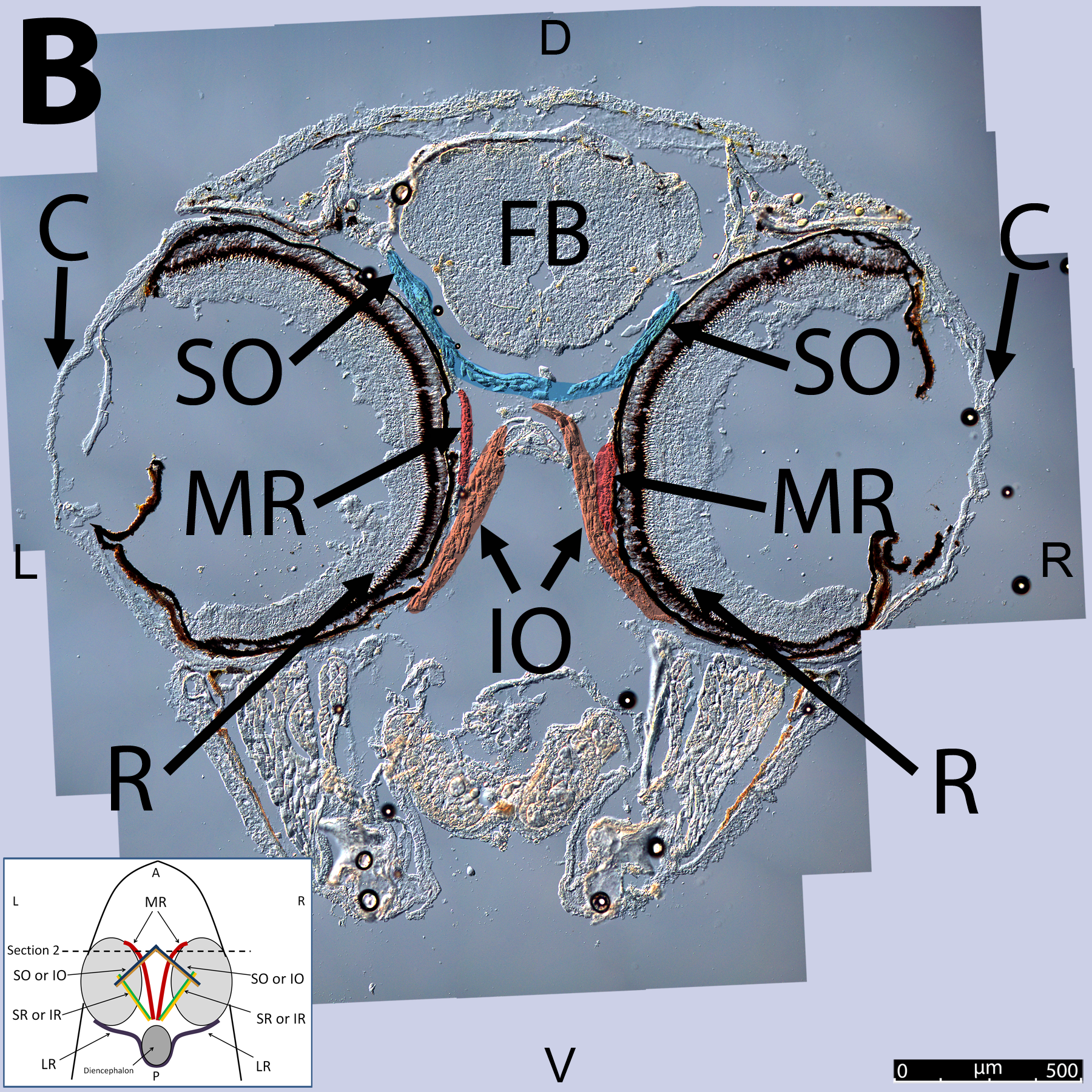

Supplement: Figure S14 [file pone.0027095.s014.tif]

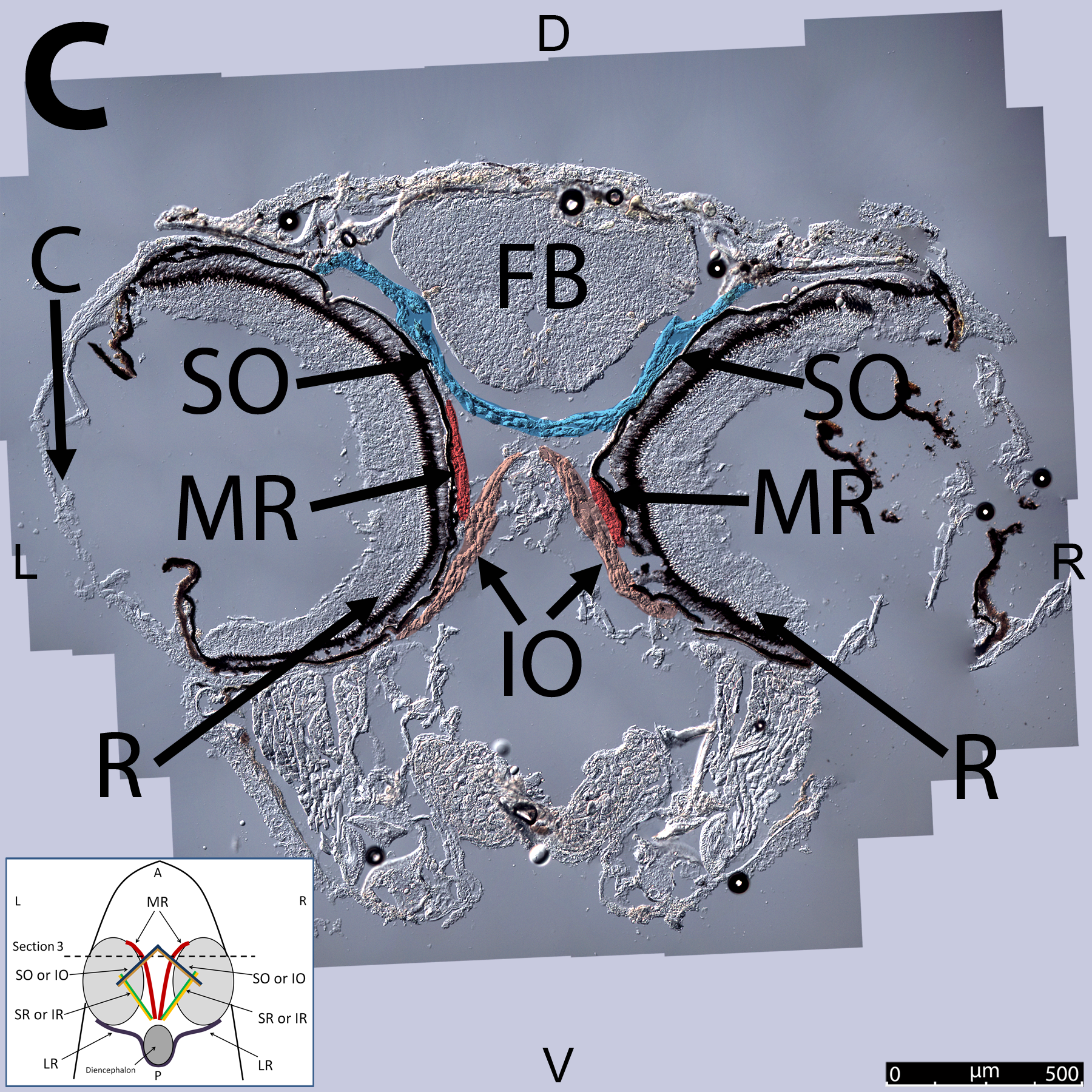

Supplement: Figure S15 [file pone.0027095.s015.tif]

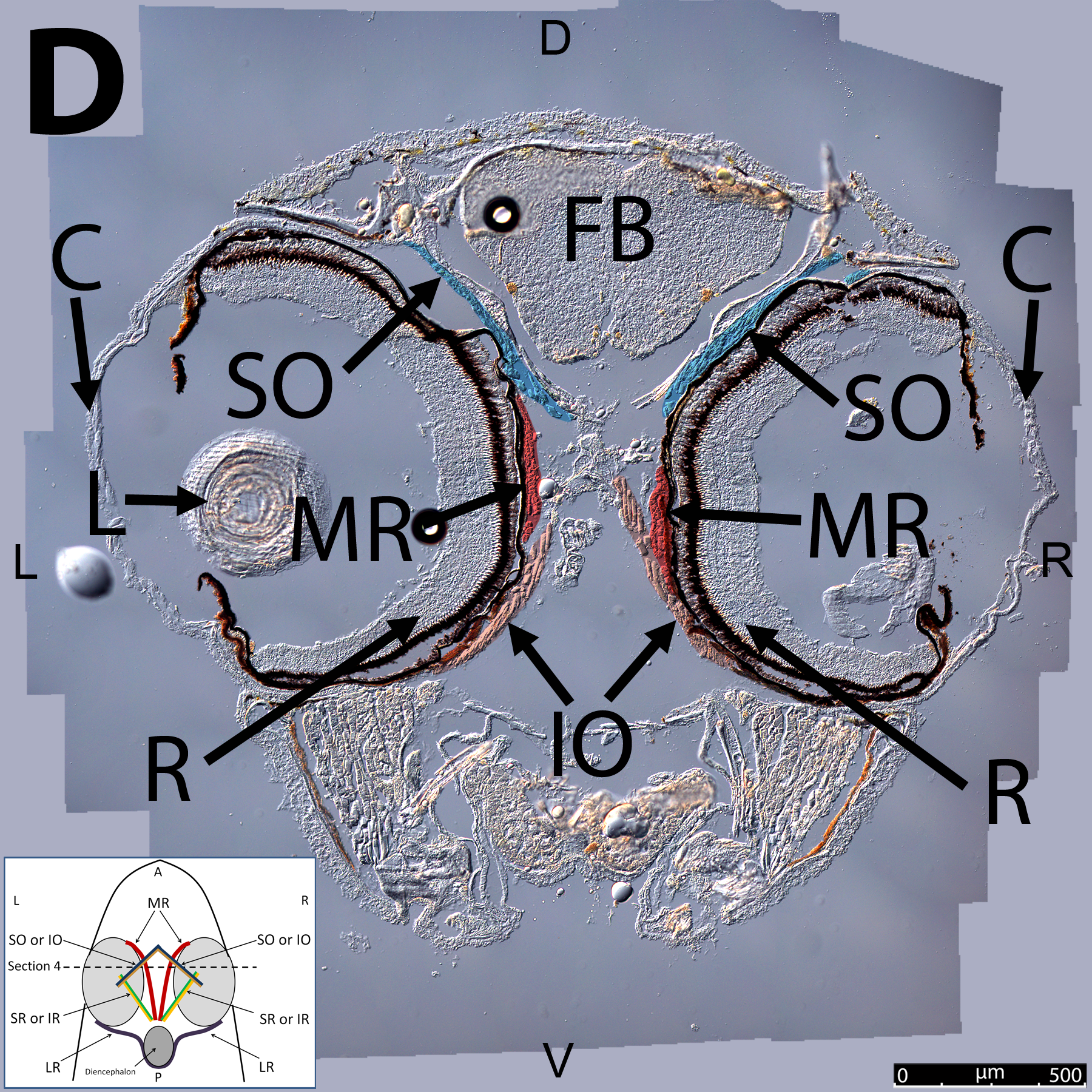

Supplement: Figure S16 [file pone.0027095.s016.tif]

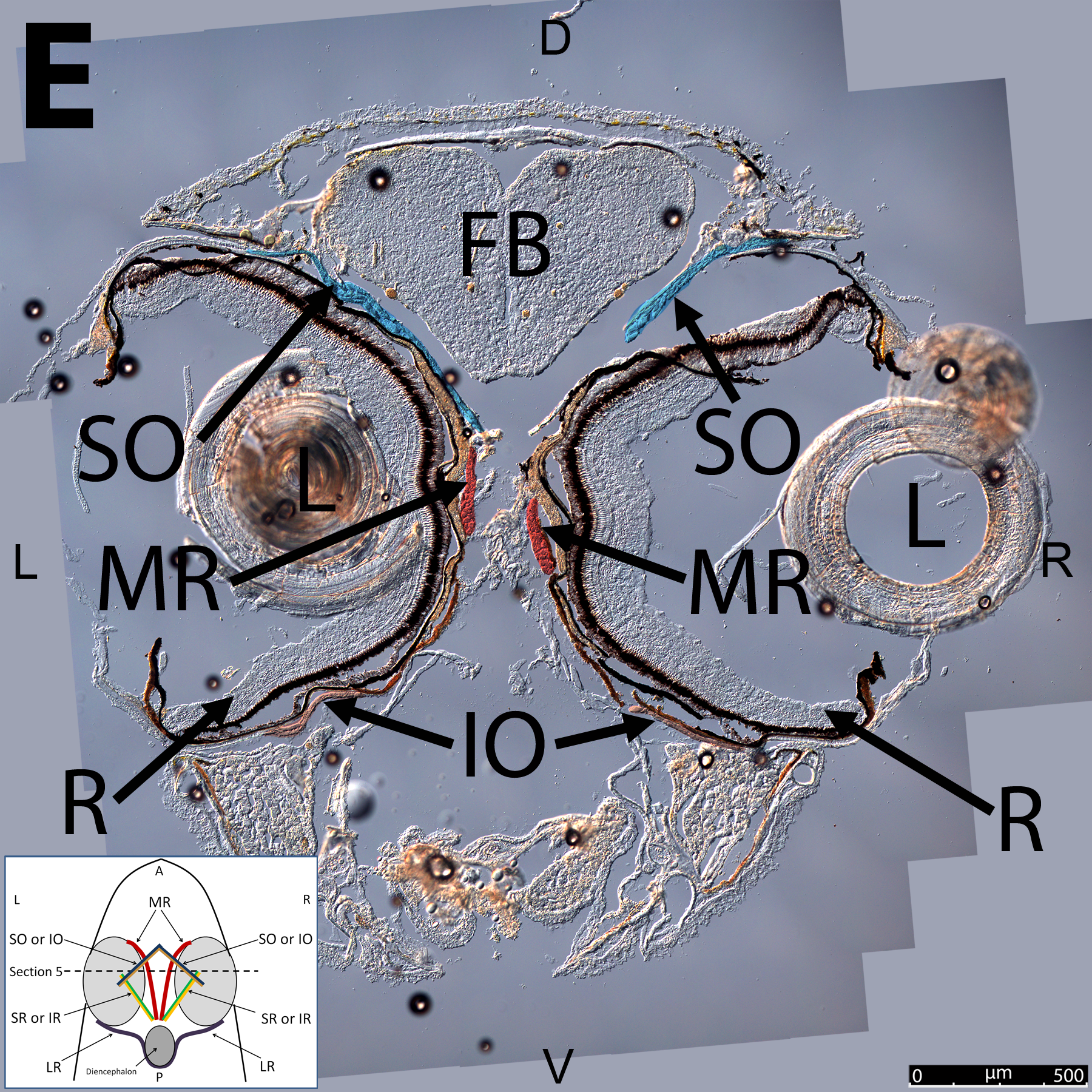

Supplement: Figure S17 [file pone.0027095.s017.tif]

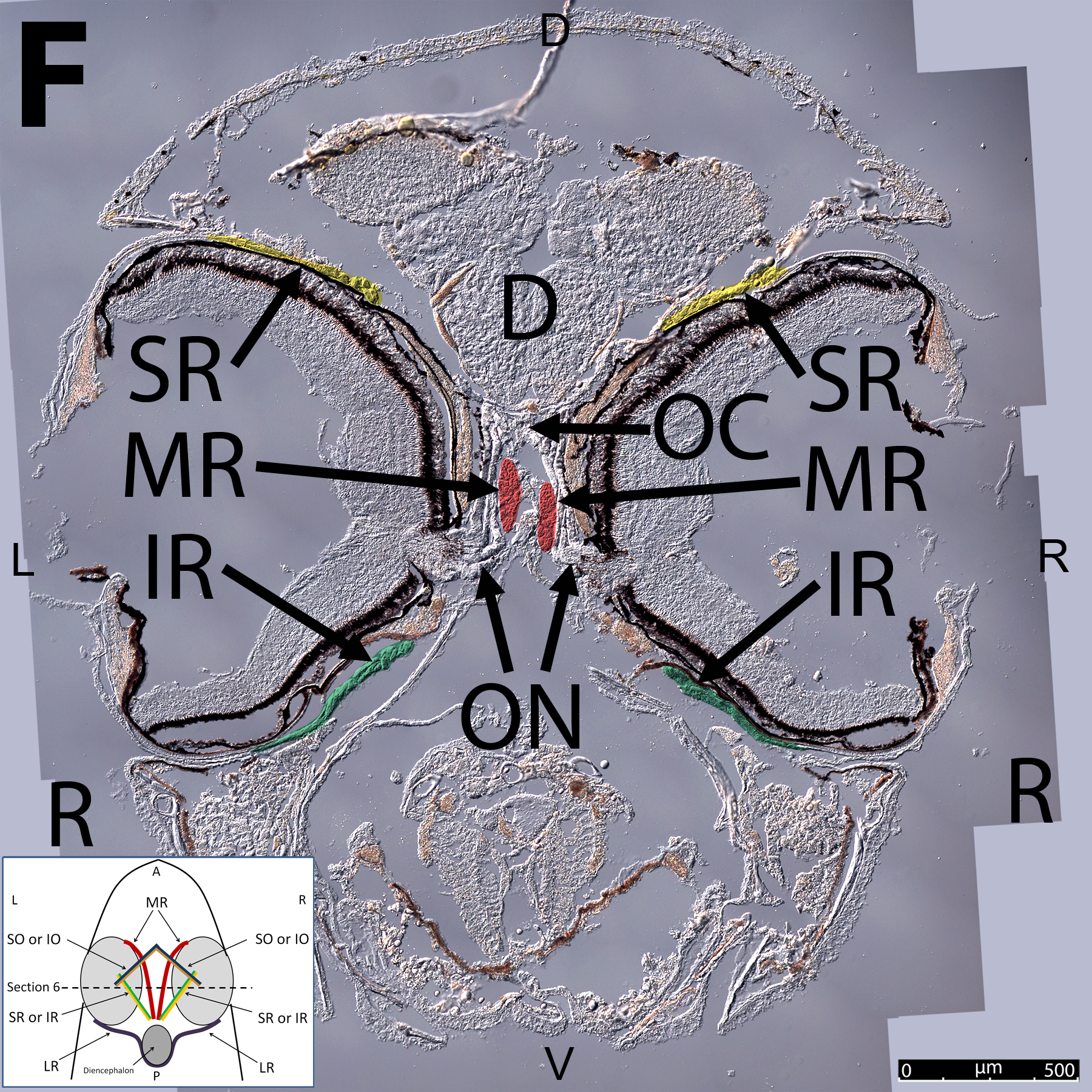

Supplement: Figure S18 [file pone.0027095.s018.tif]

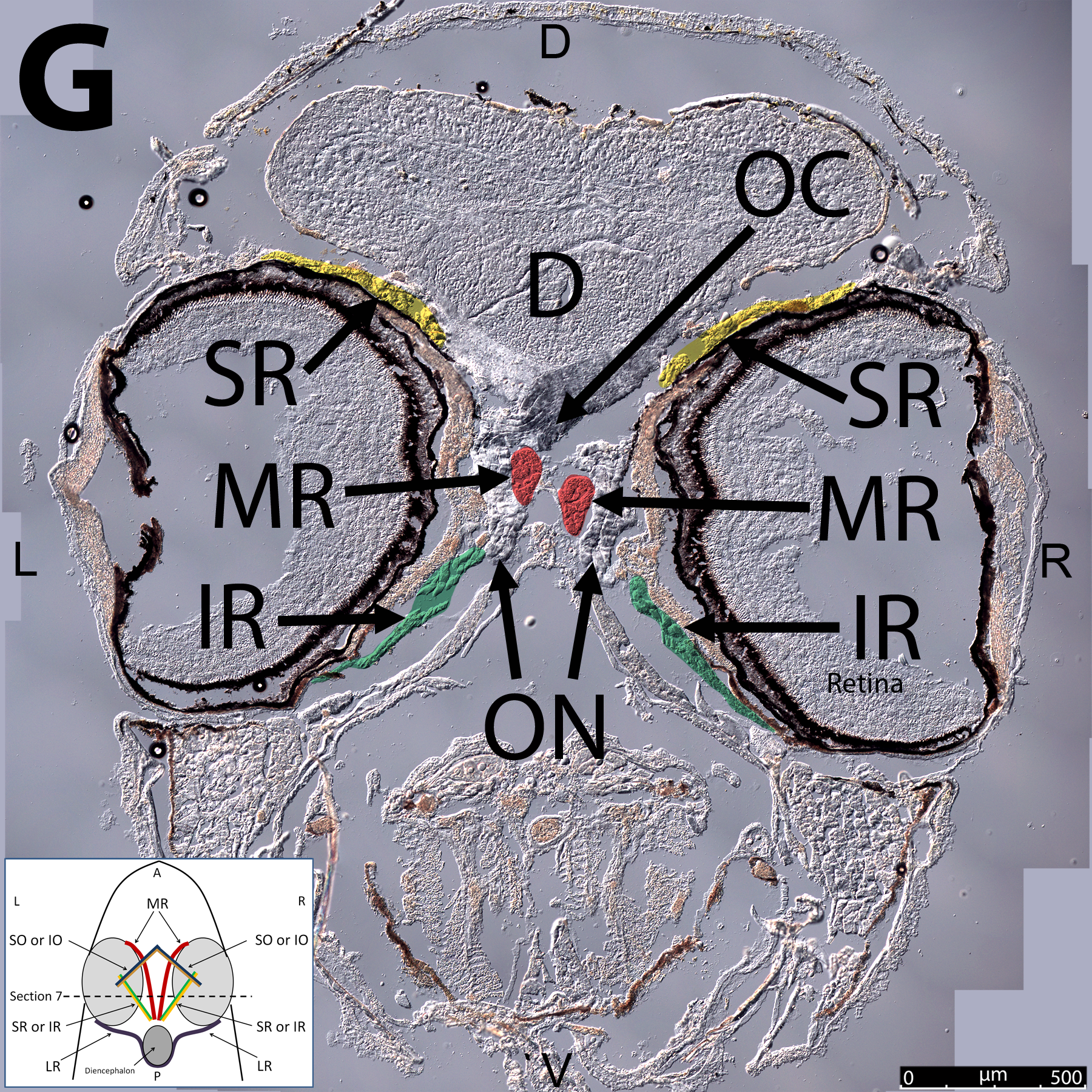

Supplement: Figure S19 [file pone.0027095.s019.tif]

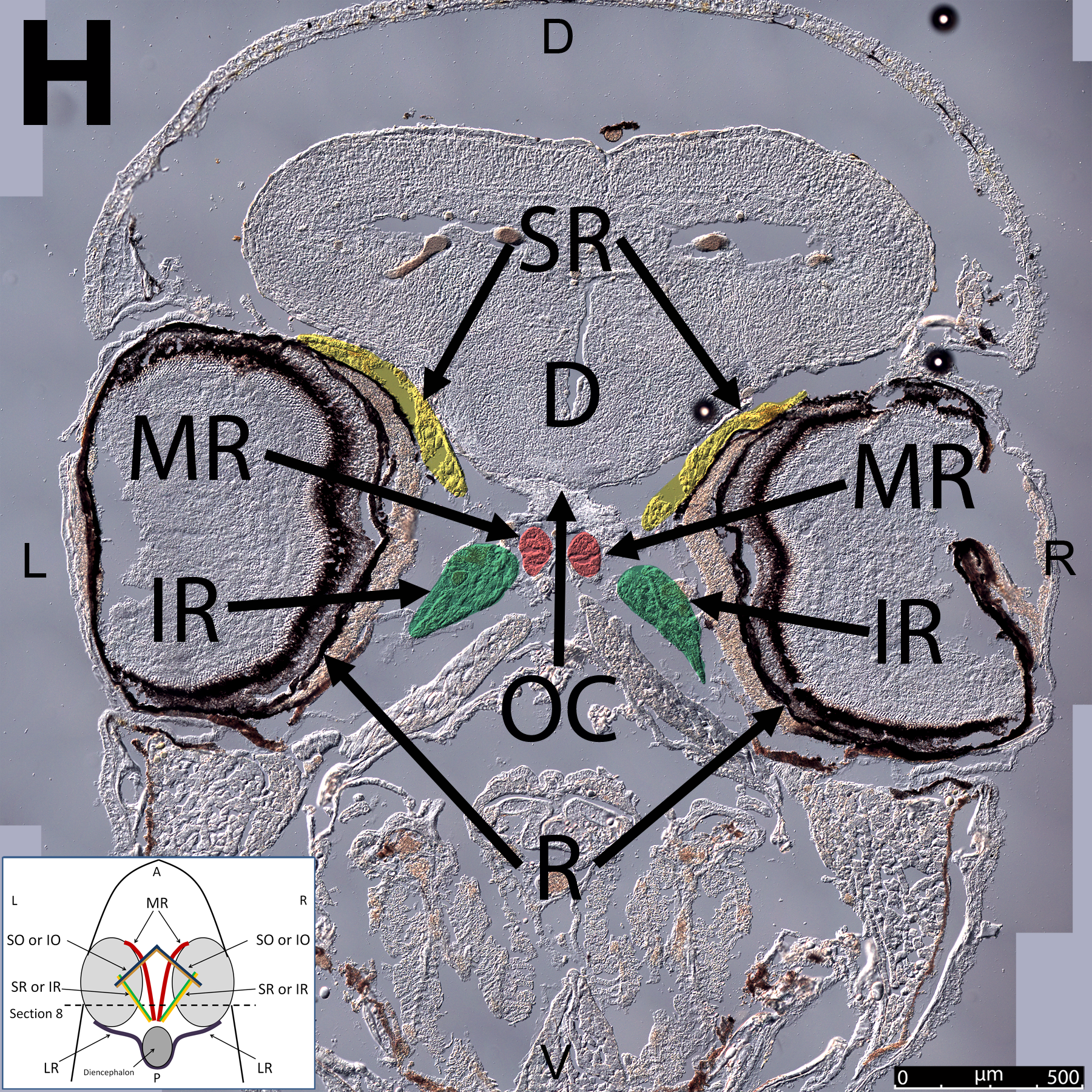

Supplement: Figure S20 [file pone.0027095.s020.tif]

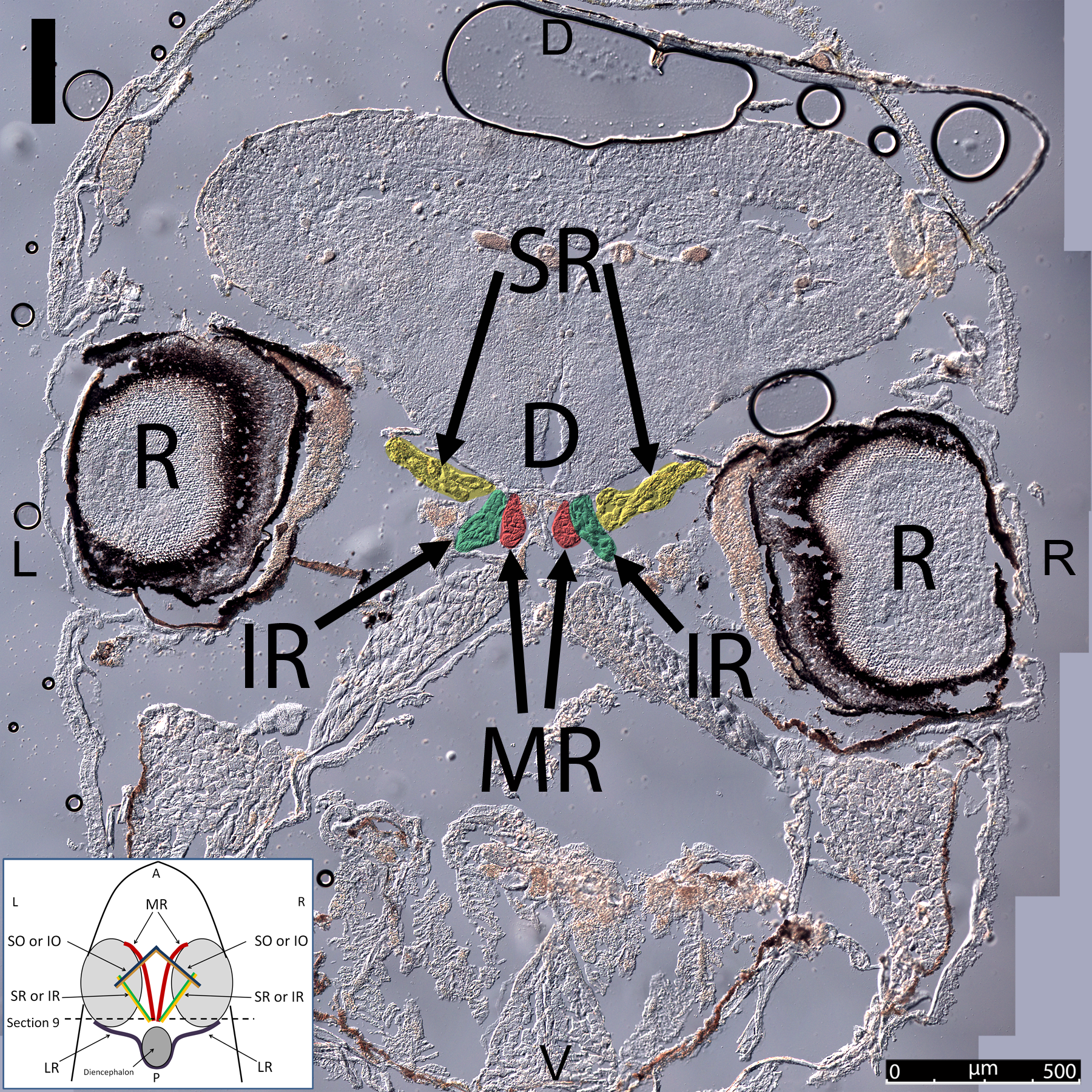

Supplement: Figure S21 [file pone.0027095.s021.tif]

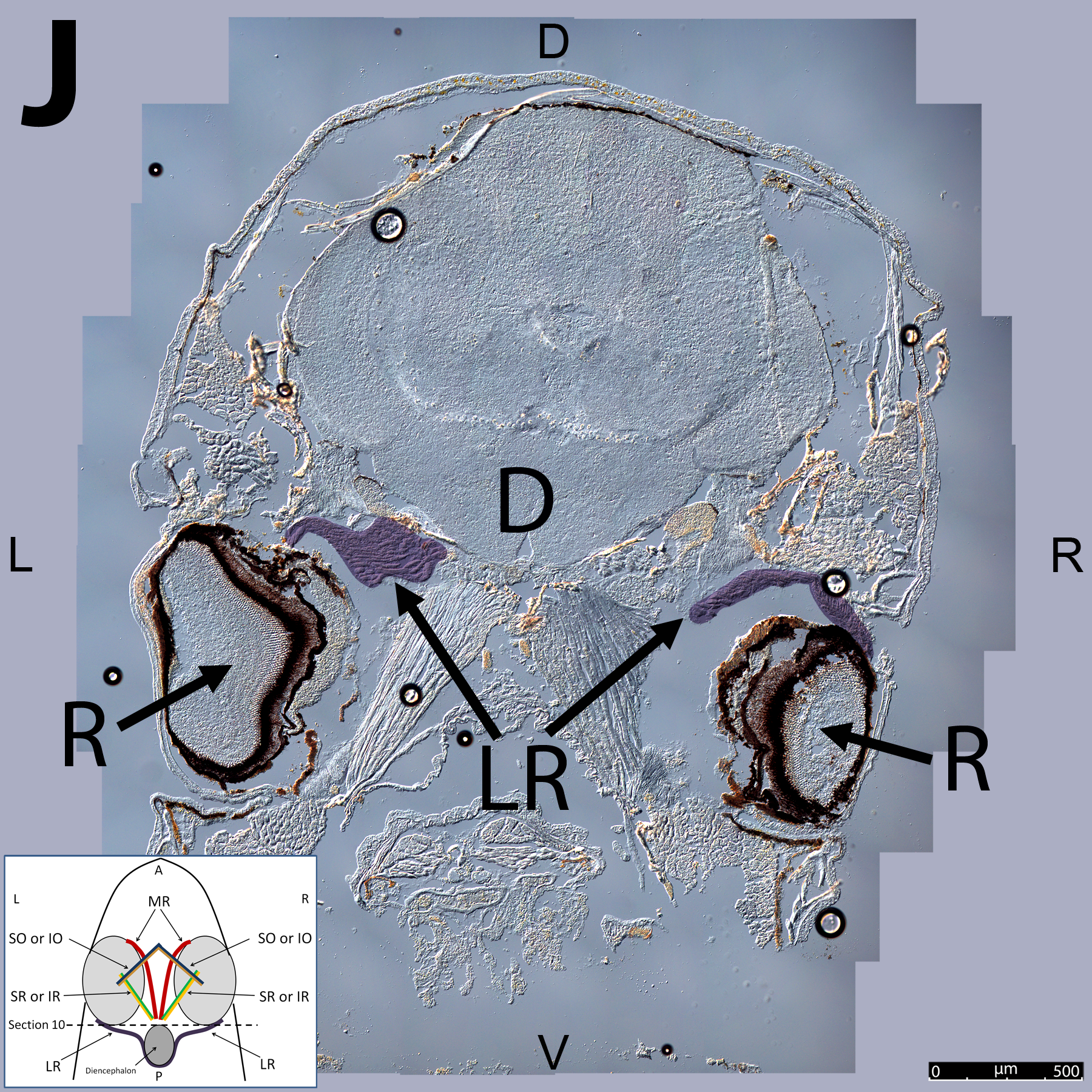

Supplement: Figure S22 — Key anatomical features within the WT adult zebrafish orbit are highlighted on 12 µm thick transverse sections originally imaged using 200X magnification with DIC prisms to show topographical tissue architecture. Sections proceed in the rostral (S13) to caudal (S22) direction and show all 6 muscles extending from origin to globe insertion. A detailed analysis of the anatomy can be found in the “Results” section of this paper. For quick reference, specific EOMs can be observed on the following figures. Superior and inferior oblique (S13–S17). Superior and inferior rectus (S18–S21). Medial rectus (S18–S21). Dorsal (D), ventral (V), left (L), and right (R) directions are noted on each frame and a schematic illustrating the specific plane of section is located in the lower left corner. (TIF) [file pone.0027095.s022.tif]
